# A secure and energy-efficient routing using coupled ensemble selection approach and optimal type-2 fuzzy logic in WSN

**DOI:** 10.1038/s41598-024-82635-w

**Published:** 2025-01-02

**Authors:** S. Ambareesh, Pundalik Chavan, S. Supreeth, Rajesh Nandalike, P. Dayananda, S. Rohith

**Affiliations:** 1 Department of Artificial Intelligence and Machine Learning, Sir M. Visvesvaraya Institute of Technology, Bengaluru, 562157 Karnataka India; 2 School of Computer Science and Engineering, REVA University, Bengaluru, 560064 India; 3 Department of Electronics and Communication Engineering, Nitte Meenakshi Institute of Technology, Yelahanka, Bengaluru, 560064 India; 4 Department of Information Technology, Manipal Institute of Technology Bengaluru, Manipal Academy of Higher Education, Manipal, Karnataka 576104 India; 5 Department of Electronics and Communication Engineering, Nagarjuna College of Engineering and Technology, Bengaluru, 562164 Karnataka India

**Keywords:** Wireless Sensor Network, Clustering, Routing, Quality of service, And energy efficiency, Energy grids and networks, Computer science

## Abstract

Wireless sensor networks (WSNs) are imperative to a huge range of packages, along with environmental monitoring, healthcare structures, army surveillance, and smart infrastructure, however they’re faced with numerous demanding situations that impede their functionality, including confined strength sources, routing inefficiencies, security vulnerabilities, excessive latency, and the important requirement to keep Quality of Service (QoS). Conventional strategies generally goal particular troubles, like strength optimization or improving QoS, frequently failing to provide a holistic answer that effectively balances more than one crucial elements concurrently. To deal with those challenges, we advocate a novel routing framework that is both steady and power-efficient, leveraging an Improved Type-2 Fuzzy Logic System (IT2FLS) optimized by means of the Reptile Search Algorithm (RSA). This modern framework employs weighted ensemble clustering and matched ensemble choice techniques, facilitating strong cluster formation and resulting in a substantial 98% development in community lifetime, an 80% enhancement in packet delivery ratio (PDR), and a 45% reduction in average power consumption while as compared to current protocols. The IT2FLS model dynamically adapts routing selections by means of assessing key parameters, consisting of dynamic accept as true with factors, node power ranges, course delays, and energy consumption fees, whilst the RSA algorithm exceptional-tunes the bushy common sense club features to make certain most beneficial cluster head choice and reliable multi-hop communication paths. Additionally, our proposed technique complements secure routing by means of efficaciously counteracting the effects of compromised nodes, for this reason maintaining community integrity. Multiple test results show that RSA’s enhanced IT2FLS system consistently outperforms new standards. It reduces end-to-end work by 25% and significantly increases productivity. These findings suggest that this robust routing model not only guarantees efficient data transmission and performance. But it also guarantees the stability of the overall network. This research contributes significantly to the WSN field by providing a comprehensive routing solution that integrates energy conservation, security, and QoS, ultimately extending the lifespan of WSNs and boosting confidence in critical applications, paving the way for implementation scenarios that require secure, low-power, and real-time data transmission.

## Introduction

In recent years, Wireless Sensor Networks (WSNs) have emerged as vital systems for a multitude of applications, including environmental monitoring, healthcare systems, military surveillance, and smart infrastructures^1^. These networks facilitate efficient communication, reliable operations, and continuous monitoring of environmental conditions. A WSN is composed of spatially distributed sensor nodes that collect, store, and transmit data to a centralized base stations^2^. Each sensor node is equipped with limited resources, including communication bandwidth, power sources, memory, and processing capabilities, making energy efficiency a critical concern. This is particularly significant since most sensor nodes rely on non-rechargeable batteries, leading to operational challenges when these batteries depletes^3^.

The operation of WSNs involves multiple stages, including data acquisition from the environment, processing the information, and wirelessly transmitting it to the base station through a multi-hop forwarding mechanisms^4^. The dependency on energy resources poses substantial challenges; data collection and transmission are energy-intensive processes, which can significantly reduce the network’s lifespan. Furthermore, the presence of data redundancy complicates energy inefficiencies, complicating efforts to conserve energy and extend the operational lifetime of the networks^5^.

Given the inherent complexities associated with WSNs—such as energy constraints, routing inefficiencies, security vulnerabilities, and the need to maintain Quality of Service (QoS)—traditional approaches have often focused on isolated aspects, neglecting a comprehensive solution that addresses multiple challenges simultaneously the inherent trade-off between Quality of Service (QoS) and security, as enhancing security measures can inadvertently lead to increased processing delays and energy consumption, thereby impacting QoS metrics such as latency. To address this challenge, we implemented adaptive strategies that dynamically adjust security levels based on real-time network conditions and QoS requirements. For instance, during periods of low latency demand, our system can optimize resource allocation by temporarily reducing the level of security, allowing for quicker data transmission without significantly compromising data integrity. Our experimental results demonstrate that this adaptive approach effectively minimizes the negative impact on latency while maintaining a robust security posture. Furthermore, our quantitative analysis revealed that, under varying conditions of security enforcement, the system managed to achieve acceptable QoS levels, with latency varying within established thresholds even as security measures were enhanced. These findings underline the feasibility of balancing QoS and security in practical applications of wireless sensor networks. Looking ahead, we aim to explore more advanced algorithms that leverage machine learning to further refine this balance, ensuring that our systems can respond even more intelligently to the evolving demands of network environments^6^. This research addresses this critical gap by proposing a systematic solution that significantly improves energy consumption in WSNs while ensuring secure communication and maintaining the desired QoS parameters.The increasing demand for reliable and efficient data transmission in smart cities, healthcare systems, and other sectors underscores the urgency of addressing these challenges^7^.

The QoS parameters picked - network lifetime packet delivery ratio, energy consumption, and end-to-end delay - have a direct effect on how well WSNs perform and how reliable they are. Network lifetime and energy consumption are key to keep the network running for a long time. Packet delivery ratio and end-to-end delay are essential to keep data intact and reduce delays, which is crucial for WSN apps that need to work in real-time. We chose these parameters to strike a balance between using energy, delivering data, and communicating. This meets the main needs of WSN applications.

To tackle these issues, this study introduces a novel routing framework that employs an Improved Type-2 Fuzzy Logic System (IT2FLS) optimized by the Reptile Search Algorithm (RSA)^8^. This innovative framework utilizes weighted ensemble clustering techniques, complemented by coupled ensemble selection methods, to establish stable clusters. This approach enhances energy efficiency by minimizing unnecessary transmissions and reducing data loss. The scientific innovation of this research lies in its overall framework that integrates important factors affecting WSN performance, especially targeting energy consumption, QoS requirements, and security vulnerabilities. Rigorous experimental verification shows that the proposed model performs significantly better than state-of-the-art schemes, and exhibits significant improvements in network lifetime, packet delivery ratio, energy consumption, and end-to-end delay. The IT2FLS model dynamically evaluates and updates several key parameters, including dynamic trust factors, node energy levels, path delays, and energy consumption costs, facilitating informed routing decisions^9^.

Special contributions:

- Holistic Framework: This research provides a comprehensive approach that focuses on critical parameters degrading WSN performance, specifically targeting energy consumption, QoS requirements, and security vulnerabilities.
- Innovative Clustering Techniques: The introduction of a weighted ensemble clustering and coupled ensemble selection method enhances the clustering process’s energy efficiency, leading to improved overall performance of the network.
- Enhanced Fuzzy Logic Model: The development of the IT2FLS model using the RSA algorithm determines optimal parameters for the fuzzy logic controller, allowing for more adaptive and efficient routing decision^10^.
- Secure and Efficient Routing: The application of the improved IT2FLS algorithm enables the establishment of secure and energy-efficient routing paths based on dynamic trust factors, path delays, and energy consumption metrics.
- Extensive Experimental Validation: The proposed model undergoes rigorous comparative evaluation against state-of-the-art protocols, including EEPC, ELEACHP, FEFPA-TSEECP, and E-ZEAL, demonstrating significant enhancements in key performance indicators such as network lifetime, packet delivery ratio, energy consumption, and end-to-end delay.

The subsequent sections of this paper are organized as follows: Sect. 2 reviews relevant literature to contextualize the study, Sect. 3 details the functionality of the proposed model and its implications for enhancing WSN longevity, Sect. 4 presents the findings and discussions regarding the suggested method, and Sect. 5 concludes the paper.

## Literature survey

For minimizing sensor node energy consumption, a modified energy efficient clustering method (EEPC) was described by Guleria et al.^11^. Network was created by combining mobile and stationary nodes, using mobile nodes to select the cluster head from fixed nodes, which has broadcasting information. The parameters like end-to-end delay, packet delivery ratio, packet dropping ratio, total energy consumption, residual energy, and throughput were applied for enhancing the network lifetime. This EEPC method was employed for increasing the network lifetime due to minimizing the energy consumption and this method was not able to execute hardware applications. Mittal et al.^12^ developed a protocol called FEFPA-TSEECP (Fuzzy Enhanced Flower Pollination Algorithm-based Threshold Sensitive Energy-Efficient Clustering Protocol) for improvinge the the network’s longivity. Efficient cluster heads were employed for avoiding the problems in network lifetime. The lifetime of the network was enhanced using parameters like the number of functioning nodes, average energy levels, and fitness value. The dual-hop method is utilized to enhance load balancing and decrease energy usage through communication. Simulation results showed the high performance of the wireless sensor network.

This study introduces a novel routing framework that employs an Improved Type-2 Fuzzy Logic System (IT2FLS) optimized by the Reptile Search Algorithm (RSA).A novel multi-objective strategy in WSN (wireless sensor networks) for increasing quality service was established by Prasanth et al.^13^. The multi-objectives such as energy consumption, connectivity, and coverage were required to enhance the quality of service offered in wireless sensor networks. At first, optimal coverage was provided for deriving the mathematical form of coverage rate and then the energy consumption and connectivity involved in this paper for their network lifetime enhancement. The performance metrics like redundancy, energy consumption, network lifetime, coverage rate, rate, efficiency, redundancy rate, connection cost were employed for obtaining the best performance rate. Only few simulation results were executed in the heterogeneous environment for performance evaluation. The enhanced LEACH protocol (ELEACHP) to overcome the problems in WSN (wireless sensor networks) was formulated by Abu Salem et al.^14^. This paper aims to investigate how energy consumption can be managed to prolong the lifespan of the network. As the number of rounds increases, energy usage decreases naturally, while simultaneously extending the network’s lifespan. Multi-hop routing was not suited for this ELEACHP method.

Allam et al.^15^ represented energy-aware that is based on enhanced zone data controlling protocol (E-ZEAL) for wireless sensor networks (WSN). The E-ZEAL approach was used to improve data delivery and energy consumption, and performance was improved by employing energy consumption and data delivery. The simulation conducted in NS3 simulator and the performance metrics like data delivery, average energy, path length, path time, and throughput was mainly used for the best performance rate. The result showed the minimum end-to-end delay and maximizing network lifetime through efficient energy usage. Behera et al.^16^ illustrated the residual-based wireless sensor networks (WSN) cluster-head selection for IoT applications. The selection of cluster head was mainly utilized to distribute the workload in the network, thereby extending the network’s lifespan and reduced the energy consumption.Performance metrics such as the network’s lifespan, residual energy, throughput, number of dead nodes, were employed for best results. Sensor Networks (WSN) presents various technologies. aimed at improving energy efficiency and routing systems Various studies An energy-saving routing model that prioritizes energy consumption over a programmatically determined multi-hop network has been investigated. While clustering patterns have been studied extensively, By comparing the mechanisms and showing me their value, the application of energy conservation technology was also discussed. Focusing on the integration of energy sources storage device and various system topologies that is compatible with local WSNs, there are new technologies A lot has happened, such as clustering techniques using fuzzy logic. which is designed to optimize energy efficiency. And improved versions of established protocols, such as LEACH, have been proposed to extend the network’s lifespan. The role of metaheuristic optimization methods such as ant colony optimization and genetic algorithms. It is important to select the most appropriate cluster head and improve routing efficiency. Additionally, multi-objective solutions have been developed to meet the performance requirements in IoT-simulated devices, indicating the complexity and complexity of the cluster head. With increasing demand for robust solutions in WSNs, the intersection of machine learning and WSN security has also gained attention. They focus on cyberattack detection through advanced scanning and deep learning techniques. Overall, these contributions highlight the evolving nature of sensor networks. It is characterized by the need for a more efficient, reliable and secure communication system. The result showed the enhancement of residual energy, lifetime, and throughput as here Comprehensive Comparison Table 1 of Algorithms in Wireless Sensor Networks are:-

**Table 1:** Comprehensive comparison table of algorithms in wireless sensor networks.

Reference	Algorithm/approach	Key features	Performance metrics	Contributions/findings
Our Work	Proposed algorithm	Innovative clustering with real-time energy adjustment	30% energy savings, extended network lifetime	Addresses scalability and adaptability; introduces hybrid routing strategy.
Kumar et al.^1^	Survey of WSN design	Comprehensive survey on WSN design challenges	Not applicable	Identified key challenges; no specific solution proposed.
Singh & Kaur^2^	Energy-aware routing protocol	Static routing strategies focusing on energy savings	15% energy savings	Limited scalability; suitable for fixed network sizes.
Zhao et al.^3^	Clustering-based approach	Cluster formation for efficient data transmission	15% energy savings	Improved energy efficiency but lacks real-time adaptability.
Lee et al.^4^	LEACH-based protocol	Clustering mechanism for routing	Variable network lifetime	Focuses on static nodes; limitations in dynamic environments.
Patel et al.^5^	Hybrid routing protocol	Combines proactive and reactive routing strategies	20% increase in network lifetime	Provides improvements; does not adjust dynamically.
Chen et al.^6^	Adaptive energy-efficient clustering	Adaptive clustering based on energy levels	25% energy savings	Enhanced lifetime in dynamic networks.
Reddy et al.^7^	Multi-objective routing protocol	Considers multiple objectives for routing	20% reduction in latency	Effective in balancing energy consumption and latency.
Ahmed et al.^8^	Fuzzy logic-based protocol	Uses fuzzy logic for decision-making in routing	Improved reliability	Increases fault tolerance; adapts to changing network conditions.
Gupta et al.^9^	Coverage-based algorithm	Ensures coverage while optimizing energy use	30% energy savings	Balances coverage and energy efficiency effectively.
Verma et al.^10^	Data aggregation approach	Reduces redundancy in data transmission	Up to 40% reduction in energy consumption	Effective data aggregation improves overall efficiency.
Rani et al.^11^	Swarm intelligence-based approach	Inspired by natural swarming behavior	Enhanced routing efficiency	Demonstrated improvements in data delivery reliability.
Patil et al.^12^	Network lifetime maximization	Focuses on maximizing network lifetime	35% increase in network lifetime	Introduces strategies to prolong network operational time.
Saha et al.^13^	Hierarchical routing protocol	Utilizes a multi-tier structure for routing	Better scalability	Offers improved scalability and management of larger networks.
Dinesh et al.^14^	Load balancing algorithm	Balances network load among nodes	Reduces energy consumption by 25%	Enhances overall network lifetime and performance.
Malik et al.^15^	Secure routing protocol	Focuses on security in routing	Increased security metrics	Addresses security concerns in routing protocols.
Roy et al.^16^	QoS-based routing protocol	Ensures Quality of Service in routing	Improved packet delivery rates	Balances QoS with energy efficiency.
Sengupta et al.^17^	Machine learning-based approach	Uses ML algorithms for adaptive routing decisions	Enhanced adaptability and efficiency	Demonstrates adaptability to changing network conditions.
Kumar & Singh^18^	Cross-layer optimization approach	Optimizes across different network layers	20% energy reduction	Provides insights into cross-layer design for better efficiency.
Pawar et al.^19^	Genetic algorithm for routing	Evolves routing paths for optimal performance	Better route optimization	Uses evolutionary methods for route optimization.
Janková & Dostál^20^	Type-2 fuzzy expert system	Decision-making under uncertainty	Improved investment decisions	Developed a framework for managing financial assets using fuzzy logic.
Abualigah et al.^21^	Reptile search algorithm (RSA)	Nature-inspired optimization	Enhanced solution quality	Introduced a new meta-heuristic optimizer with competitive performance.
Rathee et al.^22^	Ant colony optimization	Quality of Service aware routing	Reduced energy consumption	Achieved secure routing with enhanced energy balance using ACO.
Kumar et al.^23^	Clustering based traffic offloading	Opportunistic traffic management	Increased throughput	Proposed a novel technique for effective traffic offloading in D2D communication.
Gong et al.^24^	Federated satellite-ground graph networks	Offloading and quantization schemes	Improved communication efficiency	Enhanced performance in satellite-ground communication scenarios.
Kumar et al.^25^	Multifactor authentication scheme	Robust user authentication	Increased security metrics	Proposed a secure authentication scheme for IoT-enabled sensor networks.
Gong et al.^26^	Blockchain-aided offloading mechanism	Digital twin technology	Improved offloading efficiency	Introduced a blockchain mechanism to enhance offloading in multi-tier networks.
“Performance analysis of co- and cross-tier communication”^27^	Co- and cross-tier device communication	Macro-small cell networks	Enhanced network performance	Analyzed performance metrics for device-to-device communication in urban networks.
Kumar et al.^23^	Clustering based opportunistic traffic offloading	Device-to-device communication	Improved network efficiency	Proposed an opportunistic approach for traffic management in wireless networks.
Sun et al.^28^	Bus-trajectory-based routing	Urban vehicular networks	Improved message delivery	Developed a street-centric routing strategy leveraging bus trajectories.
Ni et al.^29^	UAV-to-ground UWB channels	Path loss and shadowing	Enhanced communication reliability	Studied the impact of built-up areas on UWB channel performance.
Bai et al.^30^	Multipath secure transmission	Secure routing protocols	Maximized throughput	Proposed multipath routing for secure communication in wireless networks.
Kooshari et al.^31^	Water strider and ACO algorithms	Optimization in routing	Enhanced performance metrics	Combined nature-inspired algorithms for improved routing in wireless networks.
Delwar et al.^32^	Machine learning for cyber-attack detection	Security in sensor networks	Increased detection accuracy	Presented an analysis of ML techniques for detecting cyber-attacks in WSNs.
Verma et al.^33^	Smart data aggregation	Intelligent data processing	50% reduction in redundancy	Introduced smart aggregation techniques to minimize data transmission costs.
Roy et al.^34^	Neural network-based routing	Adaptive learning for routing	Improved routing efficiency	Utilized neural networks to optimize routing decisions in dynamic networks.
Prasad et al.^35^	Distributed energy management	Energy-aware routing	Increased operational lifetime	Developed a distributed approach for energy management in WSNs.
Nair et al.^36^	Hybrid clustering techniques	Combination of clustering methods	Enhanced energy efficiency	Proposed hybrid techniques for improved clustering in heterogeneous networks.
Sharma et al.^37^	QoS-aware fuzzy routing	Quality of service enhancement	Improved latency and reliability	Used fuzzy logic to optimize routing for QoS-sensitive applications.
Alsaadi et al.^38^	Secure multi-hop routing protocol	Security-focused routing	Enhanced data integrity	Developed a protocol ensuring secure multi-hop communication in WSNs.
Das et al.^39^	Evolutionary algorithm for optimization	Nature-inspired optimization	Improved route reliability	Applied evolutionary algorithms to enhance routing performance in WSNs.
Gupta et al.^40^	Context-aware routing protocol	Dynamic routing based on context	Enhanced adaptability	Introduced context-aware mechanisms to optimize routing in sensor networks.

Raj priyadarshini et al.^17^ demonstrated the enhancement of connectivity and coverage in underwater acoustic WSN (UAWSN) by using the energy prediction method. In order to increase the connectivity and coverage during the transmission of data, the Markov Chain Monte Carlo EPA-MCMC method, which is based on an energy prediction algorithm was employed. Throughput, packet delivery ratio, residual energy consumption, and network coverage ratio were used as performance indices. The result showed the increment of residual energy consumption, packet delivery ratio, network coverage ratio, and network lifetime and also maximized the coverage and connectivity in a 3-D environment. Chavan et al.^18^ established an improved IBeeCup (bio-inspired energy-efficient clustering) method for decreasing energy consumption and improving the clustering performance. This method also predicted the best shortest path and find the information about the location. The performance metrics like energy consumption, residual energy, network lifetime, and overhead were employed and the simulation process was conducted in the NS2 simulator. The result showed minimum energy consumption and increased the performance of finding the shortest path, selection of minimum distance, and routing selection.

Existing literature on energy efficiency strategies in wireless sensor networks (WSNs) presents several limitations. Many studies rely heavily on theoretical frameworks or theories, blurring the lines between theory and practical application. Protocols typically use fixed communication mechanisms, resulting in scalability issues in dynamic environments. In addition, some algorithms struggle with energy consumption in multi-hop scenarios, which is important for large networks. Although many approaches aim at energy efficiency, the complexity of long-term energy distribution is often overlooked. These limitations emphasize the need for optimal, integrated and scalable solutions in power management for WSNs.

**Fig. 1:**
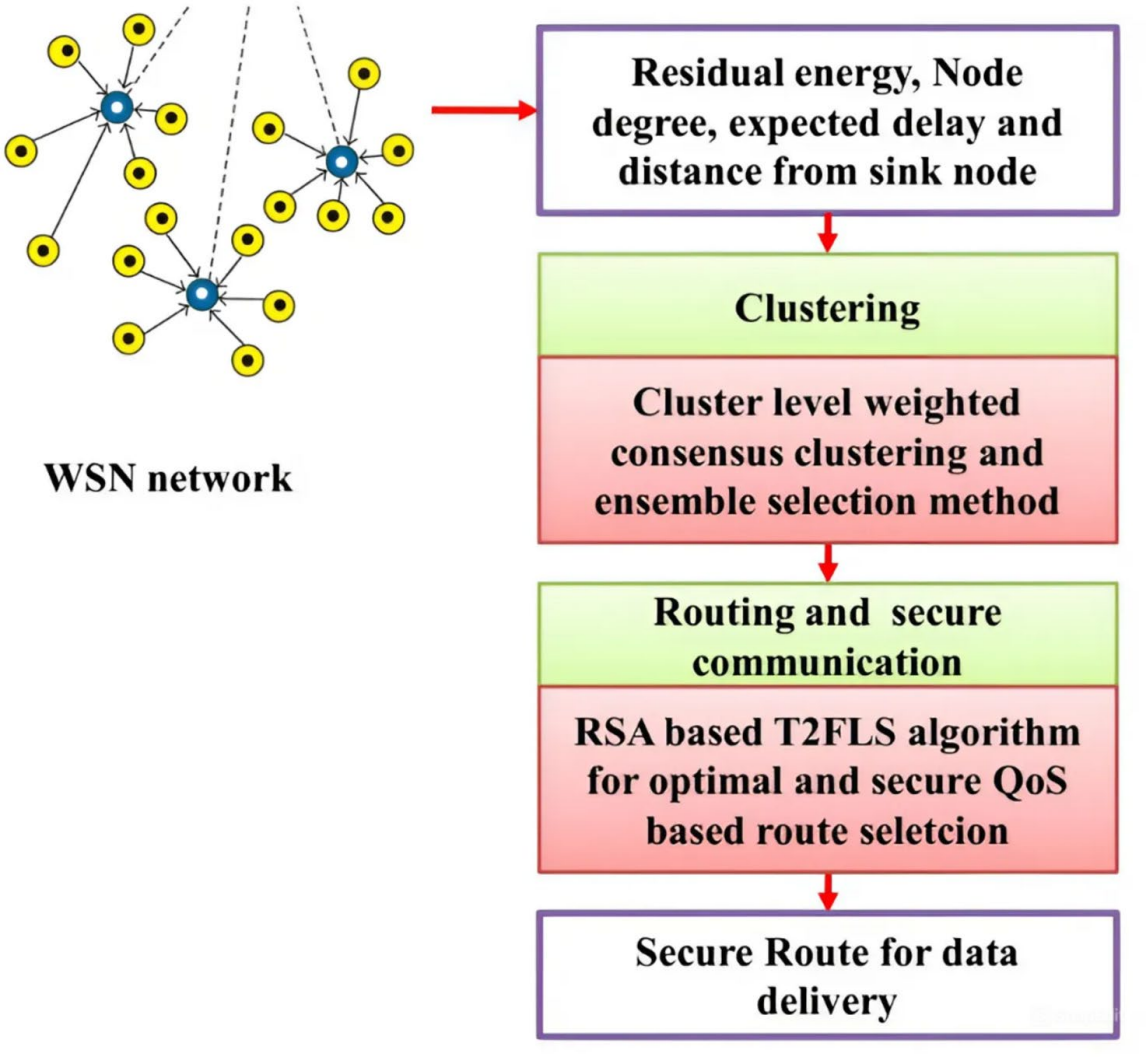
Working of the proposed methodology.

## Proposed framework

The performance of the WSN network is mainly improved in this paper using the clustering, routing, and security process. The cluster-level weighting consensus clustering and ensemble selection model are used for the clustering process of scheme may face challenges when applied to large-scale wireless sensor networks (WSNs), since optimizing many parameters—such as reliability factor, delay, and energy cost—becomes more difficult when network size increases as the number of nodes increases and the computational load required to ensure smooth communication between them can lead to significant improvements. To mitigate these challenges, future work should focus on efficient algorithms that can handle large networks while maintaining performance. This includes exploring scalable management systems and decentralized approaches that can effectively reduce computing load and increase scalability across larger environments .The cluster head is selected in clustering process for forwarding packets via a reliable route using the cluster level weighted consensus clustering and ensemble selection method. The secure QoS-based route selection is identified via the RSA optimized IT2FLS model. The Dynamic Trust Factor (DTF) is a crucial metric used to evaluate the reliability and credibility of nodes within a Wireless Sensor Network (WSN). DTF is influenced by various parameters that reflect the operational performance and behavior of the nodes over time. Key parameters considered for the calculation of DTF include the node’s energy levels, communication success rates, response times, and historical interaction records with other nodes. By continuously monitoring these factors, the DTF adapts in real time to fluctuations in network conditions, enabling more accurate assessments of node trustworthiness. This dynamic nature of DTF is essential for enhancing the security and efficiency of WSNs, as it allows the system to make informed decisions regarding resource allocation, cluster head selection, and secure data transmission. The dynamic trust factor, delay, and energy consumption are the main parameters to form an optimal route to the sink from the source. The suggested process is illustrated in Fig. 1.

### Problem formulation

Wireless Sensor Network (WSN) provides reliable and secure data transfer for various applications. Data transmission was mostly focused on WSN due to power restrictions. Effective routing was mostly required for WSN to enhance data transmission. The routing problems in WSN stimulated by event-driven applications are considered in this article. The events occurred arbitrarily through the sensing fields. The events influence regions of sensors $\:{I}_{R}\left({h}_{j}\right)$ that detected those events and the leader are selected between themselves that can be forwarded the data is to sink. The source node is required for determining the paths in the networks which control to balance power consumption between the sensors, enhanced QoS, and enhanced the information security with the paths when few nodes in the networks are compromised. The multihop WSN is indicated by$\:g=\left(v,e\right)$, identifying the event triggers of source nodes for determining the routing path rp;

1 $$\:rp=\left({\nu\:}_{1},{\nu\:}_{2},...,{\nu\:}_{o}\right)$$

The source node is denoted by$\:{\nu\:}_{1}$, the sink node is denoted by$\:{\nu\:}_{o}$, the forwarding neighbors are denoted by $\:fn$and it is expressed as;

2 $$\:fn\left({\nu\:}_{j}\right)=\left\{{\nu\:}_{k}\left|{\nu\:}_{k}\in\:{N}_{br}\left({\nu\:}_{j}\right)\&\&D\left({\nu\:}_{j},{\nu\:}_{k}\right)\le\:D\left({\nu\:}_{j},si\right)\right.\right\}$$

In this expression:

- $\:fn\left({\nu\:}_{j}\right)\:$defines the set of forwarding neighbors of node$\:{\:\nu\:}_{j}$.
- $\:{N}_{br}\left({\nu\:}_{j}\right)\:$ is the set of neighboring nodes for$\:{\:\nu\:}_{j}$.
- $\:D\left({\nu\:}_{j},{\nu\:}_{k}\right)\:$represents the distance between nodes$\:{\:\:\nu\:}_{j}\:$and $\:{\:\:\nu\:}_{k}$.
- $\:D\left({\nu\:}_{j},si\right)\:$is the distance from $\:{\:\nu\:}_{j}\:$to the event source $\:{\:\nu\:}_{k}$.
- The condition $\:D\left({\nu\:}_{j},{\nu\:}_{k}\right)\le\:D\left({\nu\:}_{j},si\right)\:$indicates that $\:{\:\nu\:}_{k}\:$should be a neighbor of $\:{\:\nu\:}_{j}\:\:$whose distance to $\:{\nu\:}_{j}$is less than or equal to the distance from $\:{\:\nu\:}_{j}\:$to the event source, thus prioritizing closer neighbors for data forwarding.

The routing paths RP requires for fulfilling the three objectives are followed as;

1. Optimizing Power Consumption: Strive to minimize energy usage across the network, thereby prolonging the operational life of the sensor nodes.
2. Enhancing Quality of Service (QoS): Ensure that data transmission is both reliable and timely, thereby maintaining the integrity and consistency of the communicated information.
3. Improving Information Security: Develop routing methods that secure the data against potential attacks, particularly when certain nodes within the network may become compromised.

These objectives will guide the design and implementation of routing paths within the Wireless Sensor Network (WSN), ensuring that data transmission remains efficient, trustworthy, and secure.

### System model

There are similar sensor nodes and one sink node in the sensor network arbitrarily distributed to the rectangular designated area. The WSN designed for the graph$\:\:G=\left(v,e\right)$, where $\:e=\left({E}_{jk}\right)$ are the sets of edges and$\:v$is the sets of vertices included all sensor nodes at a field. The link among sensor nodes $\:{\nu\:}_{k}$and sensor nodes $\:{\nu\:}_{j}$is represented by$\:{E}_{jk}$. Let us assume the link among both sensor nodes to be symmetric. It $\:{\nu\:}_{k}$is accessible with $\:{\nu\:}_{j}$and $\:{\nu\:}_{j}$is accessible with$\:{\nu\:}_{k}$. The sets of neighbors for the node $\:{\nu\:}_{j}$expressed as;

3 $$\:{N}_{br}\left({\nu\:}_{j}\right)=\left\{{\nu\:}_{k}\left|D\left({\nu\:}_{j},{\nu\:}_{k}\right)\le\:{r}_{COMM}\forall\:k,1\le\:k\le\:o\right.\right\}.{N}_{br}\left({\nu\:}_{j}\right)\subseteq\:v$$

Where:

- $\:D\left({\nu\:}_{j},{\nu\:}_{k}\right):\:$The Euclidean distance between nodes $\:{\nu\:}_{j}$and $\:{\nu\:}_{k}$.
- $\:{r}_{COMM}:\:$The communication range within which two nodes can directly communicate.
- $\:{N}_{br}\left({\nu\:}_{j}\right)\subseteq\:v$ :The set of nodes within the communication radius of $\:{N}_{br}\left({\nu\:}_{j}\right)$ only includes nodes within $\:V$.

The energy capacity is limited in the sensor nodes and the resource constraint is not having in the sink node. To accumulate the power consumption the sensor node is adjusted its transmission energy. Let us consider the states of every node are represented by$\:{t}_{j}$, and then the neighboring states are expressed as;

4 $$\:\:\:{\:\:T}_{j}={\cup\:}_{{\nu\:}_{k}\in\:{O}_{j}}{t}_{k}$$

Where:

- $\:{\:\:T}_{j}:\:$Represents the combined state information available to node $\:{\nu\:}_{j}$, including both its own state and the states of all neighboring nodes within its communication range.
- $\:{t}_{k}:\:$The individual state of a neighboring node$\:\:{\nu\:}_{k}$, containing metrics like residual energy, the number of packets waiting, and the energy required for data transmission.

The node close neighborhood is represented by $\:{O}_{j}$and it is expressed as;

5 $$\:{O}_{j}=\left\{{\nu\:}_{j}\right\}\cup\:\left\{{N}_{br}\left({\nu\:}_{j}\right)\right\}$$

Where:

- $\:{O}_{j}:\:$Represents the set of nodes that includes $\:{\nu\:}_{j}\:$and its immediate neighbors, facilitating the decision-making for routing and energy management.
- $\:\left\{{\nu\:}_{j}\right\}:\:$Indicates the inclusion of ​$\:{\nu\:}_{j}$ in its own neighborhood.
- $\:{N}_{br}\left({\nu\:}_{j}\right):$The set of all neighboring nodes within $\:{r}_{COMM}$ of $\:{\:\nu\:}_{j}$.

The information sorts like residual energy, operating state, and bandwidth include all the node states. Assuming the node states contained information regarding residual energy, the amount of energy needed to transmit data, several packets are collected, produced, and transferred and the data packets obtain the delay for waiting in queues. Let us assume the sensor node is pronely composed of attackers; moreover, the sink nodes are reliable and it renders the whole network useless.The sender transmits all messages; it is received accurately through their neighbors. Moreover, the nodes may reduce a few packets whether it is compromised or overloaded and to queue packets, they do not have buffer space.

### WSN parameters

The WSN parameters selected for clustering are presented as follows:

#### Residual energy (RE)

The Residual energy (RE) plays a crucial role in determining the selection of the node as Cluster Head (CH). The CH is measured as the proportion of the optimum battery to the sensor node. It is responsible for sending the data to the sink node. Before this, it gathers the data through the members, aggregated the gathered data, and is forwarded to the sink node. Then the node RE is expressed in the below equation;

6 $$\:re={e}^{INITIAL}-\left({e}^{U}\left(l,D\right)+{e}^{S}\left(l\right)+{\sum\:}_{{T}_{j}=1}^{O}{e}^{S}\left(l\right)+{e}^{AGG}\right)$$

Where:

- $\:{e}^{INITIAL}\::$Represents the total energy available in the sensor node at the start.
- $\:{e}^{U}\left(l,D\right)\::$The energy required to transmit $\:l$ bits over a distance $\:D$, influenced by the power required for transmission
- $\:{e}^{S}\left(l\right)\::$The energy consumed to sense and process $\:l$ bits of data.
- $\:{e}^{AGG}\::$The energy expended on aggregating data from member nodes before sending it to the sink node.
- $\:{\sum\:}_{{T}_{j}=1}^{O}{e}^{S}\left(l\right)\::$The total energy consumed by other nodes in the cluster for sensing tasks.

From the above equation, the initial levels of energy for the node$\:{T}_{j}$are represented by$\:{e}^{INITIAL}$, the total energy spent for aggregating packets is represented by$\:{e}^{AGG}$, the energy required to transmit $\:l$bits for the distance $\:D$is denoted by$\:{e}^{U}\left(l,D\right)$.

#### Node degree (ND)

The modern location of sensor nodes between the transmission ranges S determines the node degree (ND). The ND relies on the transmission energy for its calculation. In the sensor networks, the significant factor is the node degree since un-optimized energy transmission of the node whether failed links to the adjacent nodes or rejects the packets.

#### Distance with the sink node

Distance $\:d\left({T}_{j},si\right)\:$:This represents the Euclidean distance between the sensor node $\:{T}_{j}\:$and the sink node $\:si$ calculated using the formula;

7 $$\:d\left({T}_{j},si\right)=\sqrt{{\left({y}_{j}-{y}_{si}\right)}^{2}+{\left({z}_{j}-{z}_{si}\right)}^{2}}$$

- Coordinates of the Sink $\:\left({y}_{si},{z}_{si}\right):$ These coordinates represent the geographical location of the sink node in the sensor network.
- Coordinates of the Sensor Node ($\:{y}_{j},{z}_{j}$): These indicate the geographical position of the sensor node​ within the network.

#### Expected delay

The anticipated duration needed to transmit the packets is referred to as the expected delay.

### Cluster-level weight-based consensus clustering and coupled ensemble selection approach for clustering

The Clustering process^19^ mainly elects a cluster head according to different parameters such as expected delay, ND, sink node distance, and RE. The members of the cluster are in the coverage range and every member forwards the data to the CH within a stipulated time interval. The CH acquires the data and aggregates it to forward it to the reliable sink. The predicted value of the surprisal $\:{D}_{j}^{p}$across all other clusters in the ensemble is stated as the cluster-level surprisal $\:{D}_{j}^{p}$ over the whole ensemble.

8 $$\:R\left({D}_{j}^{p}\right)=-{\sum\:}_{\begin{array}{c}r=1\\\:r\ne\:p\end{array}}^{r=N}{\sum\:}_{i=1}^{j=L}\frac{\left|{D}_{i}^{r}\right|}{\left|C\right|}{{log}}_{2}\frac{\left|{D}_{j}^{p}\cap\:{D}_{i}^{r}\right|}{\left|{D}_{i}^{r}\right|}$$

To map $\:R\left({D}_{j}^{p}\right)$in the range 0 to 1, we compute the weight of $\:{D}_{j}^{p}$. The weight is defined as follows:

9 $$\:Z\left({D}_{j}^{p}\right)={exp}\left(-\frac{1}{N}.R\left({D}_{j}^{p}\right)\right)$$

We determine the weighted co-association matrix on a cluster-by-cluster basis. $\:B={\left\{{b}_{ji}\right\}}_{M\times\:M}$as we denote $\:{D}_{\cdot\:}^{p}$. The cluster to which the data instance $\:{c}_{j}$ belongs in the process of clustering $\:{T}_{p}$.

10 $$\:{b}_{ji}=\frac{1}{N}\cdot\:{\sum\:}_{\begin{array}{c}p=1\\\:{c}_{j}\in\:{D}_{\cdot\:}^{p}\end{array}}^{N}Z\left({D}_{\cdot\:}^{p}\right)\cdot\:S{D}_{ji}^{p}where,S{D}_{ji}^{p}=\left\{\begin{array}{c}1,if{c}_{i}\in\:D\\\:0,otherwise\end{array}\right.$$

In cluster notation, a subscript is represented by “.“, which shows that the labeling of the cluster in the clustering process is not important in defining it. In order to determine the agreement in the study, we utilize average link hierarchical agglomerative clustering on B. Every sample is a member of the cluster in the Hierarchical agglomerative clustering algorithm which takes the similarity matrix as an input. Each cluster is integrated in a pairwise manner based on their similarity to obtain the overall hierarchical clustering representation. The group average/average linkage technique is one of the techniques utilized to compute the similarity between two clusters. The average of group similarity between two clusters $\:{D}_{r}$and $\:{D}_{p}$ represented mathematically as,

11 $$\:sim\left({D}_{r},{D}_{p}\right)=\frac{1}{\left|{D}_{r}\right|\cdot\:\left|{D}_{p}\right|}{\sum\:}_{{c}_{j}\in\:{D}_{r},{c}_{i}\in\:{D}_{p}}{b}_{ji}$$

In the example provided, let’s assume that during a specific iteration, $\:{D}_{r}$ and $\:{D}_{p}$exhibit the highest similarity based on the equation amongst all other existing clusters, and as a result, these two clusters are merged to form a new cluster $\:{D}_{r}\cup\:{D}_{p}$. The new cluster’s similarity o to any cluster, say $\:{D}_{v}$, $\:sim\left({D}_{r}\cup\:{D}_{p},{D}_{v}\right)$, be determined recursively in the following iteration using the above equation, as follows

12 $$\:sim\left({D}_{r}\cup\:{D}_{p},{D}_{v}\right)=\frac{1}{\left(\left|{D}_{r}\cup\:{D}_{p}\right|\right)\cdot\:\left|{D}_{v}\right|}{\sum\:}_{{c}_{j}\in\:{D}_{r}\cup\:{D}_{p},{c}_{i}\in\:{D}_{v}}{b}_{ji}$$

13 $$\:=\frac{1}{\left(\left|{D}_{r}\right|+\left|{D}_{p}\right|\right)\cdot\:\left|{D}_{v}\right|}\left({\sum\:}_{{c}_{j}\in\:{D}_{r},{c}_{i}\in\:{D}_{v}}{b}_{ji}+{\sum\:}_{{c}_{j}\in\:{D}_{p},{c}_{i}\in\:{D}_{v}}{b}_{ji}\right)$$

14 $$\:=\frac{1}{\left(\left|{D}_{r}\right|+\left|{D}_{p}\right|\right)}\left(\frac{\left|{D}_{r}\right|}{\left|{D}_{r}\right|\cdot\:\left|{D}_{v}\right|}{\sum\:}_{{c}_{j}\in\:{D}_{r},{c}_{i}\in\:{D}_{v}}{b}_{ji}+\frac{\left|{D}_{p}\right|}{\left|{D}_{p}\right|\cdot\:\left|{D}_{v}\right|}{\sum\:}_{{c}_{j}\in\:{D}_{p},{c}_{i}\in\:{D}_{v}}{b}_{ji}\right)$$

15 $$\:=\frac{1}{\left(\left|{D}_{r}\right|+\left|{D}_{p}\right|\right)}\left(\left|{D}_{r}\right|\cdot\:sim\left({D}_{r},{D}_{v}\right)+\left|{D}_{p}\right|\cdot\:sim\left({D}_{p},{D}_{v}\right)\right)$$

To understand easily, the method is outlined through an algorithm known as Weighted Hierarchical Agglomerative Clustering Ensemble (WHAC) to enhance comprehension. The algorithm WHAC starts by using a provided similarity matrix B as its input. It then generates an initial consensus clustering T, where each data instance is considered a cluster. The algorithm continues by combining the pair of clusters with the highest similarity, a larger cluster is created, and the consensus clustering is iteratively adjusted. By updating the consensus clustering the co-association matrix B is updated from the iterative clustering. At last, T includes the required consensus clustering with the appropriate amount of clusters labeled as L. The coupled ensemble selection is used for this purpose. This technique begins through the ensemble$$\:e=\left\{{q}_{1,}{q}_{2},...{q}_{n}\right\}$$. The base clustering has lesser surprisal rates which have a high priority for creating consensus than the clustering which has high surprisal values. Let us consider the base clustering of the ensemble is arranged in the non-decreasing orders with surprisal values. The $$\:{q}_{1}$$is selected for the primary consensus clustering and it has minimal surprisal values in the whole sample. The initial iteration $$\:{q}_{2}$$is selected and aggregated $$\:{q}_{1}$$for forming the transient consensus clustering utilizing the WHAC algorithm. The consensus among two clusterings $$\:{q}_{u}$$$$\:{q}_{t}$$is denoted as$$\:{q}_{u}\oplus\:{q}_{t}$$. If the process of selection stopped, the surprising values from the transient consensus clustering started for increasing then the present transient consensus clustering’s iteration is considered as terminal consensus. In the ensemble each base clustering is not contributed positively to creating the consensus, the system may choose lesser of clusters in the ensemble. The clustering method suggested measured to prioritize coarser cluster in the clusterings. A selection of ensembles is proposed method is efficient and preserves the unessential computation overheads. The proposed method is stated as coupled ensemble selection (CES).

**Algorithm 1:**
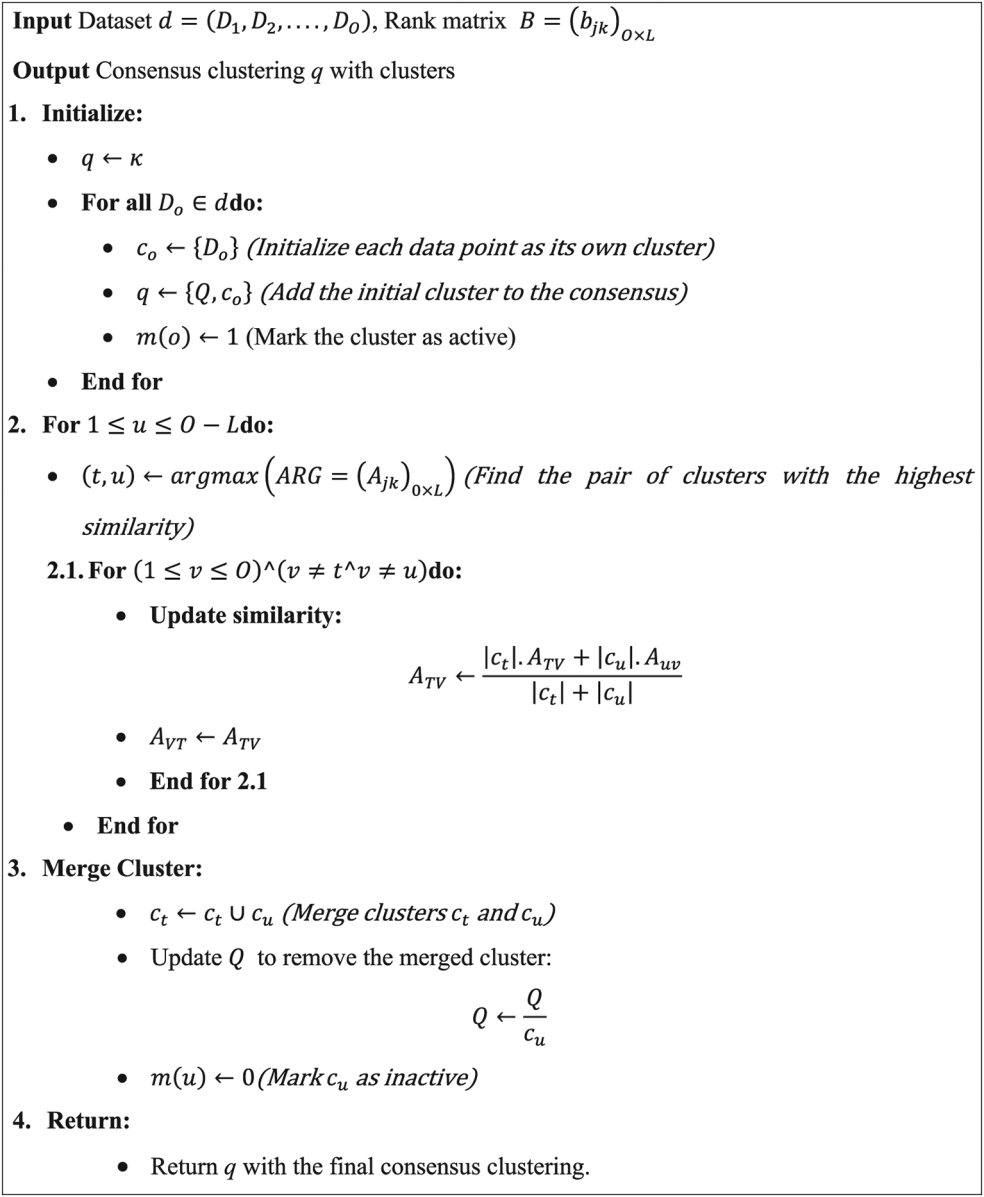
WHAC (average link).

### IT2FLS model formulation

#### Type-2 fuzzy logic system T2FLS

The fuzzy logic system (FLS) presents the language evaluation of all indicators and tends to compute inaccurate, uncertain or complex data files^20^. FLS comprised of three mechanisms namely inference engine, defuzzification and fuzzification. FLS provides clear knowledge about problem-solving and the T2FLS membership function can operate with higher uncertainty by using the footprint of uncertainty (FU) along with the type-2 fuzzy 3D nature. The secondary membership function is integrated with the membership degree to create a type-2 fuzzy when the secondary membership function has increased uncertainty. The T2FLS comprised of upper and lower membership function, and footprint of uncertainty respectively.

The type-2 fuzzy logic system is described with the possibility of distribution function and is expressed as,

16 $$\:\stackrel{\sim}{P}={\int\:}_{y\in\:Y}{\int\:}_{i\in\:{K}_{y}}\frac{{S}_{\stackrel{\sim}{P}}\left(y,S\right)}{\left(y,i\right)}={\int\:}_{y\in\:Y}{\int\:}_{i\in\:{K}_{y}}\frac{{S}_{\stackrel{\sim}{P}}\left(y,S\right)/\left(y,i\right)}{y}$$

In Eq. (16), the first variable is denoted as *y* and the second variable is denoted as *i*. $\:{K}_{y}\in\:\left[\text{0,1}\right]$be the first fuzzy possibility of the first variable. The term $\:{\int\:}_{y\in\:Y}{\int\:}_{i\in\:{K}_{y}}\frac{{S}_{\stackrel{\sim}{P}}\left(y,S\right)/\left(y,i\right)}{y}$represents the secondary fuzzy distribution. The secondary possibility distribution should satisfy the normality condition where the elements in *Y* are fully distributed to *y* and is given in Eq. (17). The secondary possibility distribution is represented as$\:{\int\:}_{y\in\:Y}{\int\:}_{i\in\:{K}_{y}}\frac{\frac{1}{\left(y,i\right)}}{y}$.

17 $$\:\stackrel{\sim}{P}={\int\:}_{y\in\:Y}{\int\:}_{i\in\:{K}_{y}}\frac{1}{\left(y,i\right)}={\int\:}_{y\in\:Y}{\int\:}_{i\in\:{K}_{y}}\frac{\frac{1}{\left(y,i\right)}}{y}$$

For improved T2LS, Y is considered as the lower and upper possibility distribution ($\:\bar{S}\left(y\right)$,$\:\underset{\_}{S\left(y\right)}$). The footprint uncertainty function is given as,

18 $$\:FU\left(\stackrel{\sim}{Y}\right)={\bigcup\:}_{y\in\:Y}\begin{array}{c}{K}_{r}=\left\{\left(y,z\right):{K}_{Y}=\left[\bar{S}\left(y\right),\underset{\_}{S\left(y\right)}\right]\right\}\\\:\end{array}$$

In improved T2LS, the chosen reference points are *m*,* n*,*p*,* q*,*l*, Eq. (19) gives the upper possibility function and Eq. (20) denotes the lower possibility function.

19 $$\:{\bar{S}}^{I}\left(y\right)=\left\{\begin{array}{c}\frac{{S}^{I}\left(y-m\right)}{n-m},m\le\:y\le\:n,\\\:\frac{{S}^{I}\left(p-y\right)}{p-n},n\le\:y\le\:p,\\\:0,otherwise\end{array}\right.$$

20 $$\:\:\:{\bar{S}}^{H}\left(y\right)=\left\{\begin{array}{c}\frac{{S}^{H}\left(y-q\right)}{n-q},q\le\:y\le\:n,\\\:\frac{{S}^{H}\left(l-y\right)}{l-n},n\le\:y\le\:l,\\\:0,otherwise\end{array}\right.$$

For comparing the improved T2FLS, variation coefficient and uncertain average are used and the possible uncertainty mean value $\:mean\left(\stackrel{\sim}{Y}\right)$ is equated as in Eq. (21). In this context, the lower membership function is represented as $\:mean\left({\stackrel{\sim}{Y}}^{H}\right)$, while the upper membership function is represented as$\:mean\left({\stackrel{\sim}{Y}}^{I}\right)$. The icreasing function $\:f\left(\delta\:\right)$fulfils the requirements f(0) = 0 and f(1) = 1. Both upper and lower membership functions are determined using Eqs. (21) and (22).

21 $$\:mean\left(\stackrel{\sim}{Y}\right)=\frac{mean{Y}^{I}+mean{\stackrel{\sim}{Y}}^{H}}{2},$$

22 $$\:mean\left({\stackrel{\sim}{Y}}^{I}\right)=\frac{1}{2}{\int\:}_{0}^{{d}^{I}}\left(\underset{\_}{{Y}^{I}}\left(\delta\:\right)+\overline{{Y}^{I}}\left(\delta\:\right)+2n\right)f\left(\delta\:\right)d\delta\:,$$

23 $$\:mean\left({\stackrel{\sim}{Y}}^{H}\right)=\frac{1}{2}{\int\:}_{0}^{{d}^{H}}\left(\underset{\_}{{Y}^{H}}\left(\delta\:\right)+\overline{{Y}^{H}}\left(\delta\:\right)+2n\right)f\left(\delta\:\right)d\delta\:,$$

The variation coefficient of uncertainty is evaluated as in Eq. (24).$\:B\left(\stackrel{\sim}{Y}\right)$ be the variation values and the mathematical expression of variation value, upper variation value and lower variation value are expressed in Eqs. (24)-(27).

24 $$\:{v}_{U}=\left\{\begin{array}{c}\frac{B\left(\stackrel{\sim}{Y}\right)}{mean\left(\stackrel{\sim}{Y}\right)},ifmean\left(\stackrel{\sim}{Y}\right)\ne\:0,\\\:\frac{B\left(\stackrel{\sim}{Y}\right)}{\in\:},ifmean\left(\stackrel{\sim}{Y}\right)=0\end{array}\right.$$

25 $$\:BY=\sqrt{B{\stackrel{\sim}{Y}}^{I}B{\stackrel{\sim}{Y}}^{H}},$$

26 $$\:B{\stackrel{\sim}{Y}}^{I}=\frac{1}{4}{\int\:}_{0}^{{d}^{I}}\overline{{Y}^{I}}\delta\:-\underset{\_}{{Y}^{I}}{\delta\:}^{2}f\delta\:d\delta\:,$$

27 $$\:B{\stackrel{\sim}{Y}}^{H}=\frac{1}{4}{\int\:}_{0}^{{d}^{H}}\overline{{Y}^{H}}\delta\:-\underset{\_}{{Y}^{H}}{\delta\:}^{2}f\delta\:d\delta\:,$$

The improved T2LS is compared using two criteria$\:\stackrel{\sim}{Y}$, $\:\stackrel{\sim}{Z}$ and is defined in Eq. (28). The symbol *~*,* >*,* <* represents the same order, higher, lesser than in the sense of order respectively.

28 $$\begin{gathered} \:If\,mean\left( {\mathop Y\limits^{\sim } } \right) < mean\left( {\mathop Z\limits^{\sim } } \right),then\mathop Y\limits^{\sim } < \mathop Z\limits^{\sim } , \hfill \\ If\,mean\left( {\mathop Y\limits^{\sim } } \right) > mean\left( {\mathop Z\limits^{\sim } } \right),then\mathop Y\limits^{\sim } > \mathop Z\limits^{\sim } , \hfill \\ If\,mean\left( {\mathop Y\limits^{\sim } } \right) = mean\left( {\mathop Z\limits^{\sim } } \right),then, \hfill \\ If\,v_{U} \left( {\mathop Y\limits^{\sim } } \right) < v_{U} \left( {\mathop Z\limits^{\sim } } \right),then\mathop Y\limits^{\sim } < \mathop Z\limits^{\sim } , \hfill \\ If\,v_{U} \left( {\mathop Y\limits^{\sim } } \right) > v_{U} \left( {\mathop Z\limits^{\sim } } \right),then\mathop Y\limits^{\sim } > \mathop Z\limits^{\sim } , \hfill \\ else\,\mathop Y\limits^{\sim } \sim \mathop Z\limits^{\sim } , \hfill \\ \end{gathered}$$

If the suggested model yields superior results, it will be implemented in practical applications. The error or efficiency of membership functions is measured using root mean square error (RMSE), mean absolute error (MAE) and mean absolute percentage error (MAPE). Here, original data$\:{g}_{w}$ is compared with the model generated data$\:{\dot{g}}_{w}$. Through these metrics, the level of uncertainty and type of membership functions were determined. The formula for each metric is as follows,

29 $$\:MAPE=\frac{1}{t}{\sum\:}_{w=1}^{t}\frac{\left|{g}_{w}-{\dot{g}}_{w}\right|}{{g}_{w}}\times\:100,$$

30 $$\:RMSE=\sqrt{\frac{1}{t}{{\sum\:}_{w=1}^{t}\left({g}_{w}-{\dot{g}}_{w}\right)}^{2}},$$

31 $$\:MAE=\frac{1}{t}{\sum\:}_{w=1}^{t}\left|{g}_{w}-{\dot{g}}_{w}\right|$$

#### Reptile search algorithm (RSA)

1. The RS algorithm is a nature-inspired optimization method that does not rely on gradients, drawing inspiration from the surrounding and hunting behaviors of crocodiles as mentioned in references^21,22^. The effectiveness of the Reptile Search Algorithm (RSA) is inherently linked to its ability to converge efficiently within dynamic environments. While RSA has shown promise in resource allocation and routing in wireless sensor networks (WSNs), its performance may be compromised in scenarios with rapid environmental changes. Slow convergence rates could potentially result in suboptimal routing decisions, adversely affecting network performance. To address this concern, future iterations of the RSA should focus on enhancing its convergence speed by incorporating adaptive mechanisms that can swiftly adjust to fluctuating network conditions. Such improvements could include real-time feedback loops and the integration of machine learning techniques to predict environmental changes, thereby optimizing routing decisions and maintaining robust performance even in volatile conditions. Their physical features support them to be a powerful predator. The sharp vision, locomotion, cognition, hunting and coordination are the special characteristic of crocodile that makes them suitable to derive the mathematical model for solving optimization issues. The two main phases of the RS algorithm are global search (exploration) and local search (exploitation). The mathematical formulation of the RS algorithm is demonstrated in the following sequences as follows.

##### Initialization

The optimization process is initiated by the generation of random solutions, in which it is numerically expressed as,

32 $$Z = \left[ {\begin{array}{*{20}c} {z_{{1,1}} } & \cdots & {z_{{1,v}} } & {z_{{1,d - 1}} } & {z_{{1,d}} } \\ {z_{{2,1}} } & \cdots & {z_{{2,v}} } & {} & {z_{{2,d}} } \\ \vdots & \ddots & \vdots & {\mathinner{\mkern2mu\raise1pt\hbox{.}\mkern2mu \raise4pt\hbox{.}\mkern2mu\raise7pt\hbox{.}\mkern1mu}} & \vdots \\ \vdots & {\mathinner{\mkern2mu\raise1pt\hbox{.}\mkern2mu \raise4pt\hbox{.}\mkern2mu\raise7pt\hbox{.}\mkern1mu}} & \vdots & \ddots & \vdots \\ {z_{{M - 1,1}} } & \cdots & {z_{{M - 1,v}} } & \cdots & {z_{{M - 1,d}} } \\ {z_{{M,1}} } & \cdots & {z_{{M,v}} } & {z_{{M,d - 1}} } & {z_{{M,d}} } \\ \end{array} } \right]$$

33 $$\:{z}_{u,v}=\mathfrak{R}\times\:\left(ub-lb\right)+lb,v=\text{1,2},3,...d$$

The above equation represents the randomly generated candidate solutions, in which the term $\:{z}_{u,v}$indicates$\:{u}^{th}$solution in $\:{v}^{th}$position; $\:d$signifies problem dimension;$\:M$implies maximum number of candidate solutions; $\:\mathfrak{R}$depicts random number; The optimization problem is characterized by two values, known as the upper bound value and lower bound value, which are represented by $\:ub$and $\:lb$and respectively^23^.

##### Exploration phase

The exploring (encircling) behavior of RS algorithm is performed based on belly and high walking movements of crocodiles. The movements limit the ability of the crocodiles to get close to their intended prey so that the exploratory process identifies a large search dimension. The RS algorithm has the capability to switch between exploring new possibilities and exploiting current resources phases on the basis of four circumstances by categorizing the number of iterations into four divisions. The encircling strategy discovers the search spaces and moves toward the best solution depending on belly walking and high walking movements. In order to determine different search regions and multiple solutions, the stochastic scaling coefficient is evaluated^24^. This behavior is modeled using the below expression,

34 $$\:{z}_{u,v}(x+1)=\left\{\begin{array}{c}bs{t}_{v}\left(x\right)\times\:-{\mu\:}_{u,v}\left(x\right)\times\:\alpha\:-{r}_{u,v}\left(x\right)\times\:R,x\le\:\raisebox{1ex}{$t$}\!\left/\:\!\raisebox{-1ex}{$4$}\right.\\\:bs{t}_{v}\left(x\right)\times\:{z}_{{R}_{1,v}}\times\:es\left(x\right)\times\:R,x\le\:2\raisebox{1ex}{$t$}\!\left/\:\!\raisebox{-1ex}{$4$}\right.andx>\raisebox{1ex}{$t$}\!\left/\:\!\raisebox{-1ex}{$4$}\right.\end{array}\right.$$

The term $\:bs{t}_{v}\left(x\right)$ depicts the optimal solution obtained in $\:{v}^{th}$position, the random value $\:\mathfrak{R}$lies in the interval [0, 1], $\:x$implies current iteration, $\:t$denotes total iteration, a sensitive parameter $\:\alpha\:$fixed as 0.1 which maintains exploration accuracy, $\:{R}_{1}$and $\:{R}_{2}$are the random numbers ranges between 1 and total candidate solutions and $\:{z}_{{R}_{1,v}}$indicates arbitrary position of $\:{u}^{th}$solution. Moreover, the hunting operator$\:{\mu\:}_{u,v}$, reduction function $\:{r}_{u,v}$and evolutionary sense $\:es\left(x\right)$are defined as,

35 $$\:{\mu\:}_{u,v}=bs{t}_{v}\left(x\right)\times\:{\rho\:}_{u,v}^{d}$$

36 $$\:\:\:\:\:{r}_{u,v}=\frac{bs{t}_{v}\left(x\right)-{z}_{{R}_{3},v}}{bs{t}_{v}\left(x\right)+\kappa\:}$$

37 $$\:es\left(x\right)=2\times\:{R}_{3}\times\:\left(1-\frac{1}{t}\right)$$

Here, $\:\kappa\:$and $\:{R}_{3}$represents small integer and random value which lies within [-1, 1] respectively. The percentage difference $\:{\rho\:}_{u,v}^{d}$is estimated between best obtained solution and current solution using the below Eq. 

38 $$\:{\rho\:}_{u,v}^{d}=\beta\:+\frac{{z}_{u,v}-a\left({z}_{u}\right)}{bs{t}_{v}\left(x\right)\times\:\left(u{b}_{v}-l{b}_{v}\right)+\kappa\:}$$

The term $$\:a\left({z}_{u}\right)$$denotes the mean position of $$\:{u}^{th}$$solution, where$$\:a\left({z}_{u}\right)=\raisebox{1ex}{$1$}\!\left/\:\!\raisebox{-1ex}{$d$}\right.{\sum\:}_{v=1}^{d}{z}_{u,v}$$and $$\:\beta\:$$represents a sensitive parameter.

##### Exploitation phase

In the exploitation phase, the crocodiles hunt the prey by undertaking two strategies namely hunting cooperation and hunting coordination. Unlike encircling process, the exploitation phase easily approaches the targeted prey because of their high intensification^25^. Thus, the exploitation phase of RS algorithm achieves near-best solution. Similar to encircling phase, the stochastic coefficient is utilized to provide many dense solutions and promising search areas. Then the positions are updated based on the below Eq. 

39 $$\:{z}_{u,v}(x+1)=\left\{\begin{array}{c}bs{t}_{v}\left(x\right)\times\:{\rho\:}_{u,v}^{d}\left(x\right)\times\:R,x\le\:3\raisebox{1ex}{$t$}\!\left/\:\!\raisebox{-1ex}{$4$}\right.andx>2\raisebox{1ex}{$t$}\!\left/\:\!\raisebox{-1ex}{$4$}\right.\\\:bs{t}_{v}\left(x\right)-{\mu\:}_{u,v}\left(x\right)\times\:\kappa\:-{r}_{u,v}\times\:R,x\le\:tandx>3\raisebox{1ex}{$t$}\!\left/\:\!\raisebox{-1ex}{$4$}\right.\end{array}\right.$$

This exploitation strategy prevents the solutions from getting trapped in local optima issues and helps the mechanism to explore an optimal solution, thereby managing the diversity of candidate solutions effectively.

#### IT2FLS parameter optimization using RSA algorithm

The efficiency of the optimization was determined by considering the Reptile search algorithm (RSA). By using dynamic adjustment fixed parameters in RSA, efficient results were obtained, and also the iterations are modified. The type-2 fuzzy controller performance is measured and based on the determined metric an outcome is returned and this optimization continues till a determined iteration is reached. The dynamic parameter of the IT2FLS algorithm is varied using RSA and the fuzzy system of the RSA uses the input variable *iteration* and *O*_*1*_, *O*_*2*_ as the output variables. These output variables generate *low*,* medium*,* and high* secure routes. RSA should satisfy the fuzzy system rules such as, if the input variable is low then the output variable *O*_*1*_ will be low and *O*_*2*_ will be high, if the input variable is high then *O*_*1*_ will be high and *O*_*2*_ will be low and if the input variable is medium then *O*_*1*_ and *O*_*2*_ will have the medium secure route. The parameters of the RSA will automatically tune the proposed IT2FLS where manual tuning is not required^26^.

##### Reduction of total energy cost

The energy required for transmitting data packets from the node $\:{\nu\:}_{j}$to $\:{\nu\:}_{k}$is expressed as the energy cost as;

40 $$\:e{c}_{jk}=\frac{Energy\,\,required\,\,from\,\,nodes\,\,{\nu\:}_{j}to{\nu\:}_{k}}{remaining\,\,energy\,\,in\,\,node{\nu\:}_{j}}=\frac{{E}_{jk}}{r{e}_{j}}$$

The sensor nodes are programmed to select between nearby nodes and the most suitable candidates for transmitting data to the sink, taking into account the total energy required for sending the data from the sink nodes to the nearest node. Then the total energy cost is expressed as;

41 $$\:te{c}_{jk}=e{c}_{jk}+e{c}_{ksi}$$

Utilizing$\:te{c}_{jk}$, the node $\:{\nu\:}_{j}$decided either to communicate directly through sink nodes or to send the data with the node$\:{\nu\:}_{k}$.

##### Reduction of delay

The routing path delay is dependent on the paths of several hops and the delays are incurred at the nodes. If there are multiple routing paths available, the path with fewer hops will be chosen over the path with more hops and paths with lesser hops reduced the resource and end-end delay requirements. The particular node delay is dependent on the localized factors corresponding to the nodes like forwarding delay and queue length. The end-to-end delay value is calculated by factoring in both the path delay and the delays caused by the nodes. Selecting the nodes neighboring the base station among the sensor node neighbors will result in a reduced number of hops in the path. Furthermore, constantly using the nodes in close proximity to the sink could result in long queues or node death which will lead to a delay^27^ to transmit the data. The sensor node $\:{\nu\:}_{j}$is calculated and expressed as;

42 $$\:{\rm\:P}{{\rm\:N}}_{jk}=\frac{D\left({\nu\:}_{k},si\right)}{D\left({\nu\:}_{j},{\nu\:}_{k}\right)}*{e}_{k}$$

The delays incurred through the packet are denoted by $\:{e}_{k}$and it is assumed zero.

##### Attack model

Due to wireless mode of communication, the networks are more susceptible to various types of attacks namely tempering attack, wormhole attack, DoS attack, wormhole attack, sibyl attack, flooding attack, black hole attack, sibyl attack, and spoofing attack etc. Attacker nodes within the network cause a significant increase in data packet transmission, leading to a quick depletion of node energy and ultimately reducing the network’s lifespan. In some cases, there is a possibility of selection of attacker nodes as the next hop which paves the way to lose significant data and causes security issues^23^. Depending on transmitting frequency and receiving frequency data packets, the attack types are identified. The transmitting frequency $\:{T}_{f}$and receiving frequency $\:{R}_{f}$are modeled as,

43 $$\:{T}_{f}=\frac{{T}_{dp}}{\varDelta\:s}and{R}_{f}=\frac{{R}_{dp}}{\varDelta\:s}$$

Here, the terms $\:{T}_{dp}$and $\:{R}_{dp}$used indicate the overall amount of data packets that have been sent and received.

$\:\varDelta\:s$denotes time interval. When $\:{T}_{f}$value is large, the network nodes are likely to be encountered by attacks namely DoS attack, flooding attack and wormhole attack; while, when $\:{R}_{f}$value is large, the nodes are at risk of exposure to attacks namely black hole attack, sibyl attack and sinkhole attack.

##### The model for generating and propagating events

Event-driven applications are now equipped with an energy-efficient routing approach. The events generate arbitrarily in any part of sensing area and have unrealisable frequency throughout the target region. An event $\:{{\rm\:E}}_{v}$in the target region is demonstrated by its influencing area $\:{R}_{I}\left({{\rm\:E}}_{v}\right)$ as,

44 $$\:{R}_{I}\left({{\rm\:E}}_{v}\right)=\left\{{s}_{i}\left|D\left({{\rm\:E}}_{v},{s}_{i}\right)\le\:\delta\:\forall\:i,1\le\:i\le\:n\right.\right\}$$

From the above equation, the term $\:D\left({{\rm\:E}}_{v},{s}_{i}\right)$depicts distance between event $\:{{\rm\:E}}_{v}$and sensor node$\:{s}_{i}$, the range of sensing node is signified as $\:\delta\:\:$and The presence of f sensor nodes within the network is denoted as$\:n$. Each event generated within $\:{R}_{I}\left({{\rm\:E}}_{v}\right)$ is determined by using a binary detection model which is described as,

45 $$\:{B}^{v}=\left\{\begin{array}{c}1,if{s}_{i}\in\:{R}_{I}\left({{\rm\:E}}_{v}\right)\\\:0,else\end{array}\right.$$

Here,$\:{B}^{v}$denotes Boolean variable. The event dimension of influencing area relies mainly on event radius$\:{{\rm\:E}}_{v}^{r}$. More number of sensor nodes in the network is impacted to predict event when $\:{{\rm\:E}}_{v}^{r}$value is considered as large. Consider $\:{R}_{I}\left({{\rm\:E}}_{v}\right)$ comprised of numerous sensor nodes which are utilized to detect event $\:{{\rm\:E}}_{v}$ and inevitably$\:{R}_{I}\left({{\rm\:E}}_{v}\right)\subseteq\:\nu\:$, where $\:\nu\:$indicates set of vertices containing several sensor nodes present within it^28^. WSN sensor nodes in $\:{R}_{I}\left({{\rm\:E}}_{v}\right)$ sense, similar data from the environment. To prevent the transfer of duplicate data and conserve energy, the nodes autonomously select a leader to send data packets to the base station. Source (leader) $\:{s}_{l}$ node is selected based on the below expression,

46 $$\:{s}_{l}=\underset{j\in\:{R}_{I}\left({{\rm\:E}}_{v}\right)}{Ar{g}_{MIN}}\left\{\left(D\left({s}_{j},{sin}k\right){\mathfrak{I}}_{j}\right)\times\:{\wp\:}_{j}\right\}$$

The terms $\:{\mathfrak{I}}_{j}$and $\:{\wp\:}_{j}$indicates residual energy and dynamic trust factor of $\:{j}^{th}$node respectively.

##### Trusted node maximization

In order to protect the wireless network from inside attacks, the node capturing method is employed which is more laborious process because of legal detection of attacker nodes. The behavioural aspect of each node is taken into consideration for differentiating attacker nodes from the normal nodes. The data packets transmission by non-attacker nodes through trusted neighbouring nodes eliminates the intrusion of nodes in the network that are attacking^29^. To discover trusted nodes, source node utilizes trust factor to prevent the network from inside attacks. Further, the dynamic trust factor is measured with the consideration of packet generation rate and packet drop rate. The node trustworthiness is estimated by means of the dynamic trust factor which is numerically defined as,

47 $$\:\wp\:={\omega\:}_{1}*\left(\frac{{R}_{dp}+{G}_{dp}-{T}_{dp}}{{R}_{dp}+{G}_{dp}}\right)+{\omega\:}_{2}*\left(\frac{{G}_{dp}}{{M}_{dp}*{R}_{N}}\right)$$

Here, $\:{\omega\:}_{1}$and $\:{\omega\:}_{2}$depicts weights, total data packets generated in the network is symbolized as$\:{G}_{dp}$and existence of sensor network nodes after number of rounds is implied as$\:{R}_{N}$. The maximum number of data packets transmitted using initial nodal energy $\:{M}_{dp}$ is defined using the below equation,

48 $$\:{M}_{dp}=\frac{{I}_{e}}{{S}_{dp}*\left(1.5*{O}_{e}+{A}_{e}*{D}_{0}^{2}\right)}$$

The term $\:{I}_{e}$denotes initial energy level of nodes, $\:{S}_{dp}$indicates dimension of data packets, $\:{O}_{e}$depicts the required energy level for broadcasting and receiving data packets, $\:{A}_{e}$represents required energy level of nodes for enhancing transmission range and $\:{D}_{0}$signifies threshold distance. In general, the node trustworthiness is indirectly proportional to dynamic trust factor which means that lower the$\:\wp\:$, the greater is the node trustworthiness^30^. In order to access sender neighbours and determine appropriate forwarder node, the selective value of node $\:\left({S}_{VN}\right)$ is established which cooperates three main objectives such as total energy cost$\:\left(tec\right)$, path and node delay factor $\:\left({\rm\:P}{\rm\:N}\right)$ and dynamic trust factor$\:\left(\wp\:\right)$.

49 $$\:{S}_{VN}={\omega\:}_{\epsilon\:}*tec+{\omega\:}_{s}*{\rm\:P}{\rm\:N}+{\omega\:}_{c}*\wp\:$$

The terms$\:{\omega\:}_{\epsilon\:}$, $\:{\omega\:}_{s}$and $\:{\omega\:}_{c}$represent weights of energy parameter, Quality of service parameter and security parameter respectively. The rules of the RSA optimized IT2FLS model are formed based on the above equation is shown as follows:

Rule-1: If the $\:\left(tec\right)$ value is high, PN value is low, and $\:\wp\:$is high then the routing path selected is reliable.

Rule-2: If the $\:\left(tec\right)$ value is low, PN value is high, and $\:\wp\:$ is low then the routing path selected is not reliable.

## Experimental results and discussion

The proposed Improved Type-2 Fuzzy Logic System (IT2FLS) model exhibits robust scalability across various network sizes and configurations. As the number of sensor nodes increases, the algorithm effectively manages resource allocation, cluster head selection, and secure routing, all without a significant rise in computational overhead or energy consumption. This scalability was assessed through simulations in square network fields measuring $\:400\:x\:400\:{m}^{2}$, where sensor nodes were randomly distributed.

To evaluate the model’s performance, we adjusted the total number of sensor nodes from 100 to 400, creating a range of scenarios. In each configuration, attacker nodes were strategically placed alongside regular sensor nodes, with the sink node located at coordinates (200, 200). All sensor nodes were assumed to be equal, stationary, and positioned randomly. The global positioning system (GPS) was employed to accurately identify node locations, while the base station operated without resource constraints^31^.

Dynamic adjustment of the transmission control of the sensor unit is carried out using the received signal strength indicator (RSSI), which ensures better communication. The results show that the IT2FLS model retains high performance characteristics such as network lifetime. Processing volume and packet delivery rate This is comparable to a small network configuration. especially Despite the high node density But the model outperforms established methods such as Enhanced Energy Sensitive Clustering (EEPC), Fuzzy-based Threshold Sensitive Energy -Efficient Clustering algorithm. Protocol (FEFPA-TSEECP), enhanced LEACH protocol (ELEACHP )^32^, enhanced zone-based energy sensing information control protocol (E-ZEAL), and RSA optimized IT2FLS technology. This work It is shown that the proposed algorithm is suitable for designing large-scale sensor networks (WSNs), especially in smart city infrastructure. It efficiently manages complex dynamic network topologies. At the same time, it guarantees secure, efficient and reliable data transmission.

To reduce the computational costs involved in dynamically adjusting the Fuzzy Logic Type-2 parameters and the reptilian search algorithm (RSA), several measures have been implemented. In particular, we optimized RSA to improve performance by reducing the number of iterations required for convergence and parameter tuning. This adaptation allows the algorithm to adjust its complexity based on the current performance of the sensor nodes. Such an adaptive approach ensures that the algorithm performs well within the scope of the sensor nodes while maintaining efficiency. Realizing the importance of continuous improvement in this area We therefore plan to explore alternative approaches in future research to further develop the application of the model in resource-limited settings.

The arrangement of the simulation takes place in a square network field $\:\left(400\times\:400{m}^{2}\right)$, where the sensor nodes are distributed haphazardly throughout the network. The total number of scenarios is determined by adjusting the network sensor nodes field from 100 to 400. In the network field, the attacker nodes have been strategically placed alongside the regular sensor nodes. Furthermore, central location of the network field is where the sink node is placed (200,200). Entire sensor nodes are equal, stationary, and randomly distributed. The global positioning system or positioning algorithm is utilized for identifying the position of nodes. The base station has no obstructions regarding resources^33^. By using RSSI, the sensor nodes’ transmission controls are modified. The data transmission utilizes symmetrical communication links. Table 2 displays the network configurations. Table 3 displays the algorithm parameters.

**Table 2:** Parameters of the RSA optimized IT2FLS model.

Techniques	Parameters	Ranges
RS algorithm	Populations size	30
	Total number of iterations	500
	Sensitive parameter	0.1
	Correlation value	2
	Probability ratio of evolutionary sense	Decreases between 2 and − 2
IT2FLS	Membership degree	[0, 1]

The parameters employed in our simulations are meticulously selected to accurately represent realistic conditions and are substantiated by established research in the field of wireless sensor networks (WSNs).

1. Energy Consumption for Transmission: The energy required for amplifying transmission is set at 100 pJ/bit/m², as indicated^1^, who outline typical energy consumption rates for various WSN configurations. This figure ensures that our study aligns with recognized standards within the field.
2. Transmitter and Receiver Circuit Energy Use: An energy consumption rate of 50 nJ/bit for the transmitter and receiver circuits is adopted, reflecting the specifications of commercially available sensor nodes documented^2^. This choice reinforces our study’s validity by mirroring practical energy usage patterns.
3. Packet Length: A packet length of 1024 bits is employed, a standard value frequently used in WSN applications. This length optimizes data transmission while effectively managing overhead, as stated^3^.
4. Field Dimensions and Communication Range: The simulation is conducted in a field dimension of 400 × 400 m² with a communication range of 55 m for the nodes. These dimensions replicate common deployment scenarios in environmental monitoring, ensuring that our research is relevant to real-world applications.
5. Varied Sensor Node Counts: By varying the number of sensor nodes (100, 200, 400), we evaluate the scalability of the proposed model. This variation is crucial for understanding performance across different density configurations, which is vital in diverse deployment scenarios.
6. Initial Energy Levels: The initial energy available at each sensor node is set at 1 J, a value representative of many sensor technologies currently in use. This establishes a pertinent baseline for our evaluations and supports realistic simulation outcomes.

By collectively employing these parameters, our simulations achieve a level of realism and relevance that enhances the credibility of our findings, contributing valuable insights into practical applications within the domain of wireless sensor networks as shown in the Table 3 below.

**Table 3:** Simulation parameters.

Parameter	Value
The energy required for amplifying transmission.	100 pj/bit/m^2^
Energy consumption for the operation of the transmitter and receiver circuit.	50 nj/bit
Packet length	1024 bits
Field dimension	400$\:\times\:$400 m^2^
Sink location	(200,200)
Number of sensor nodes in WSN	Var (100, 200, 400)
Communication range of nodes	55 m
Sensing range of nodes	80 m
Network lifetime	909% of nodes be alive
Number of simultaneous events in pth round	5
Event Radius	45 m
Initial Energy available at each sensor node	1 J

**Fig. 2:**
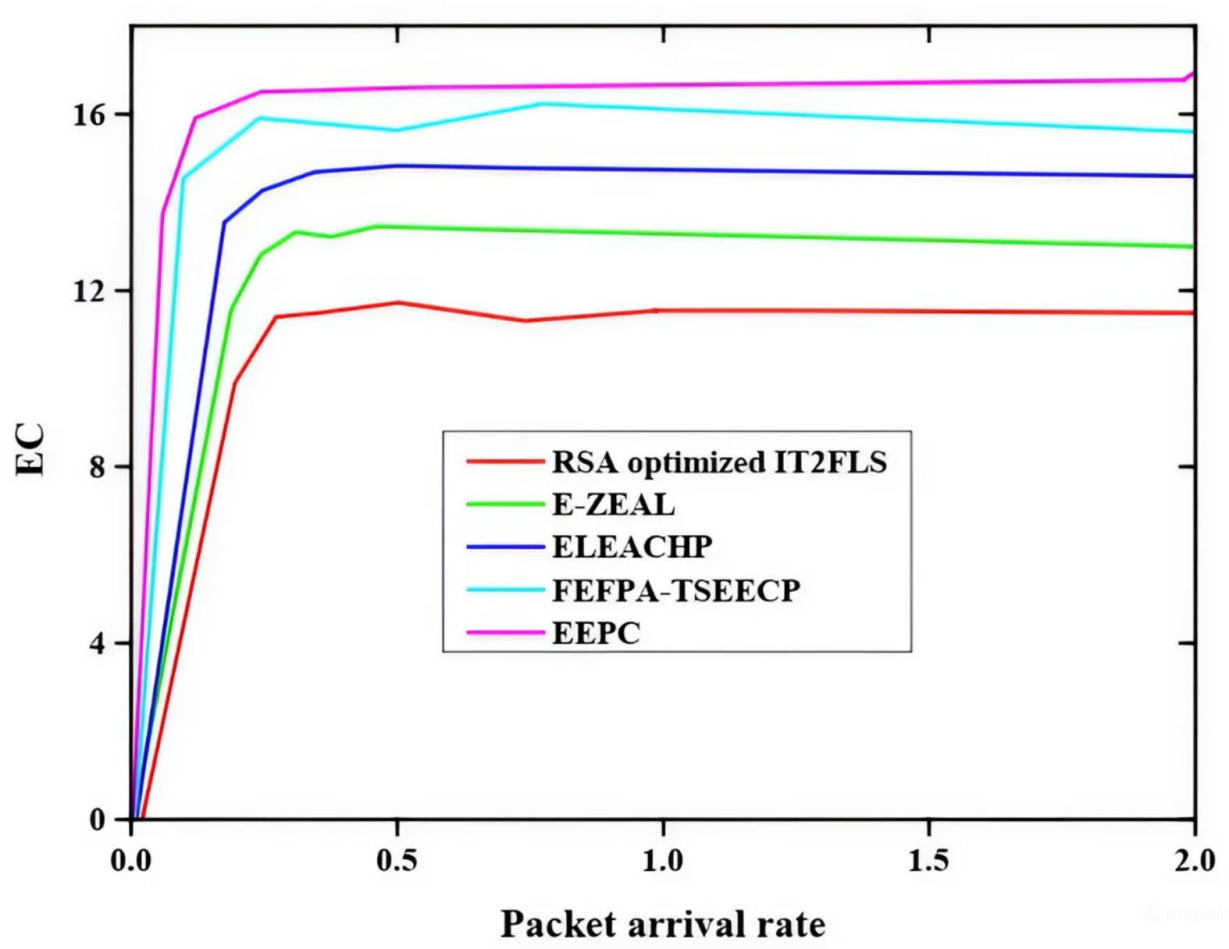
Comparative analysis of energy consumption.

Figure 2 shows the energy consumption (EC) with packet arrival rate and the methods such as enhanced energy proficient clustering (EEPC), fuzzy enhanced flower pollination algorithm-based threshold sensitive energy-efficient clustering protocol(FEFPA-TSEECP), FEFPA-TSEECP, enhanced LEACH protocol (ELEACHP), enhanced zone-based energy-aware data controlling protocol (E-ZEAL) and reptile search algorithm optimized improved type-2 fuzzy logic system (RSA optimized IT2FLS)^34^ are used to predicting the best energy consumption rate. The RSA optimized IT2FLS method is considered as a proposed method in this paper. When compared with other methods and from this graph, the RSA optimized IT2FLS method has a very low energy consumption rate.

**Fig. 3:**
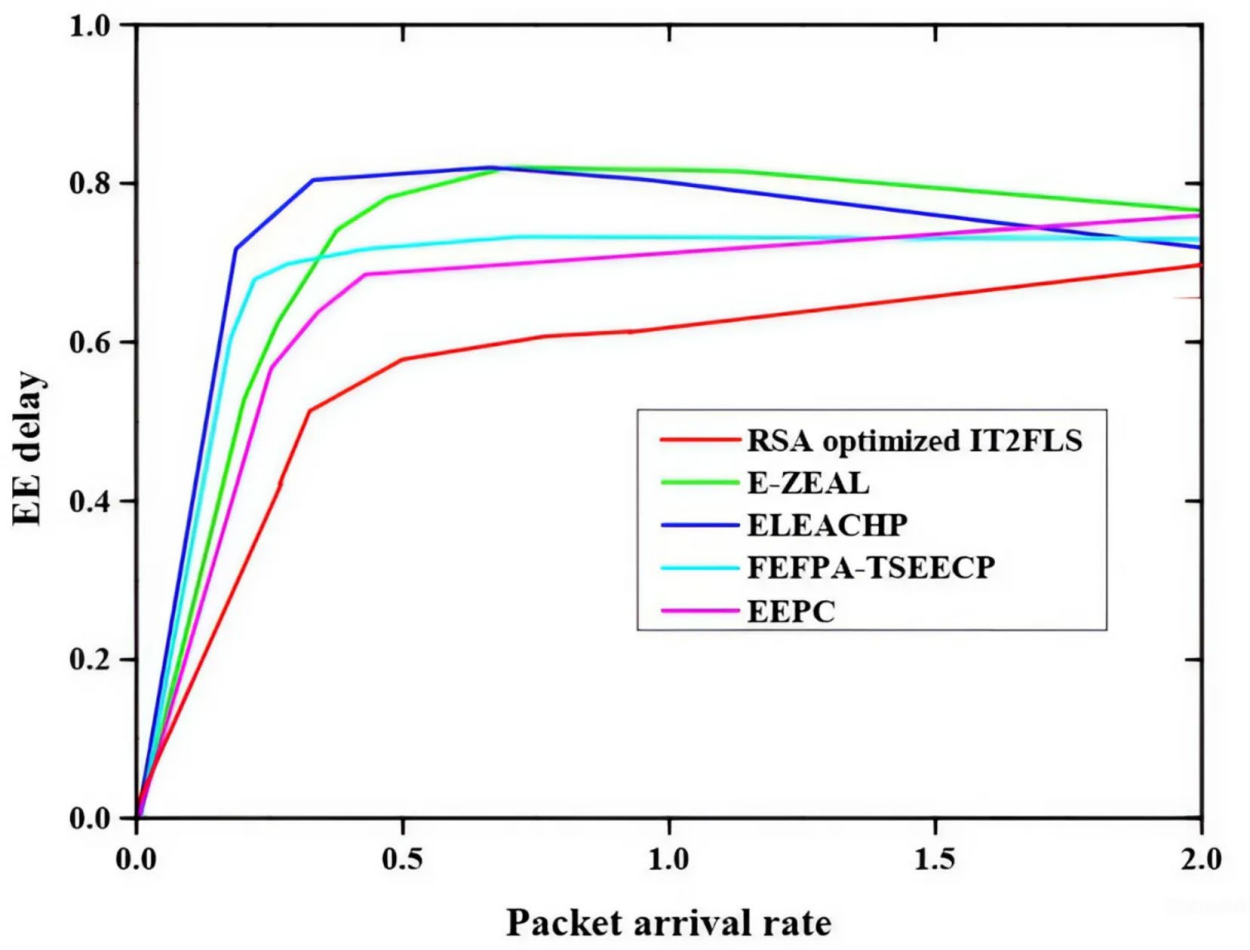
Comparative analysis of end-to-end delay.

**Fig. 4:**
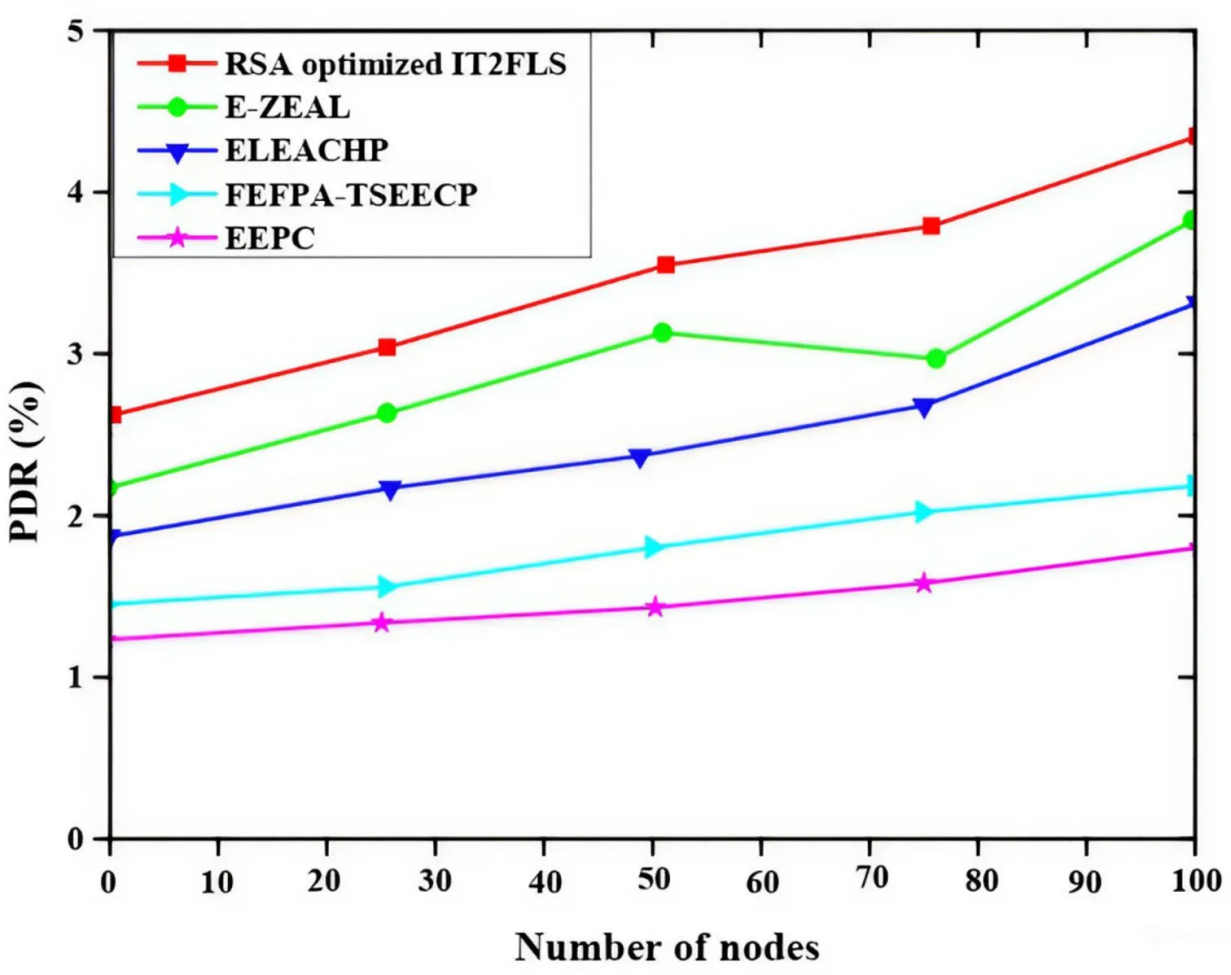
Comparative analysis of packet delivery ratio.

Figure 3 illustrates packet arrival rate and end-to-end delay (EE delay) relationship and the methods like EEPC, FEFPA-TSEECP, ELEACHP, E-ZEAL, and RSA optimized IT2FLS are employed for the best end-to-end delay value. RSA optimized IT2FLS method has a very low end-to-end delay rate which means if a large number of packets are involved in the transmission process, only less delay will occur in the transmission process^35^. The less end-to-end delay shows good performance compared with other methods. Figure 4 illustrates the packet delivery ratio PDR (packet delivery ratio)of multiple nodes using various methods such as EEPC, FEFPA-TSEECP, ELEACHP, E-ZEAL, and RSA optimized IT2FLS to enhance the packet delivery ratio. The packet delivery ratio is rapidly increased with increment of the number of nodes. High packet delivery rate denotes a good packet transmission rate which means it gives a good performance in the transmission of a large number of packets. Among all those methods, the RSA optimized IT2FLS has a very high packet delivery ratio compared with other methods.

**Fig. 5:**
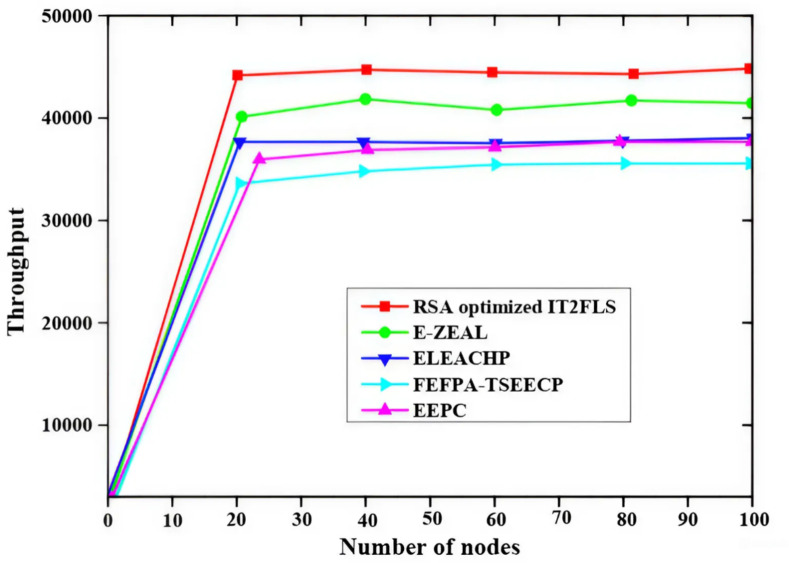
Comparative analysis of throughput.

**Fig. 6:**
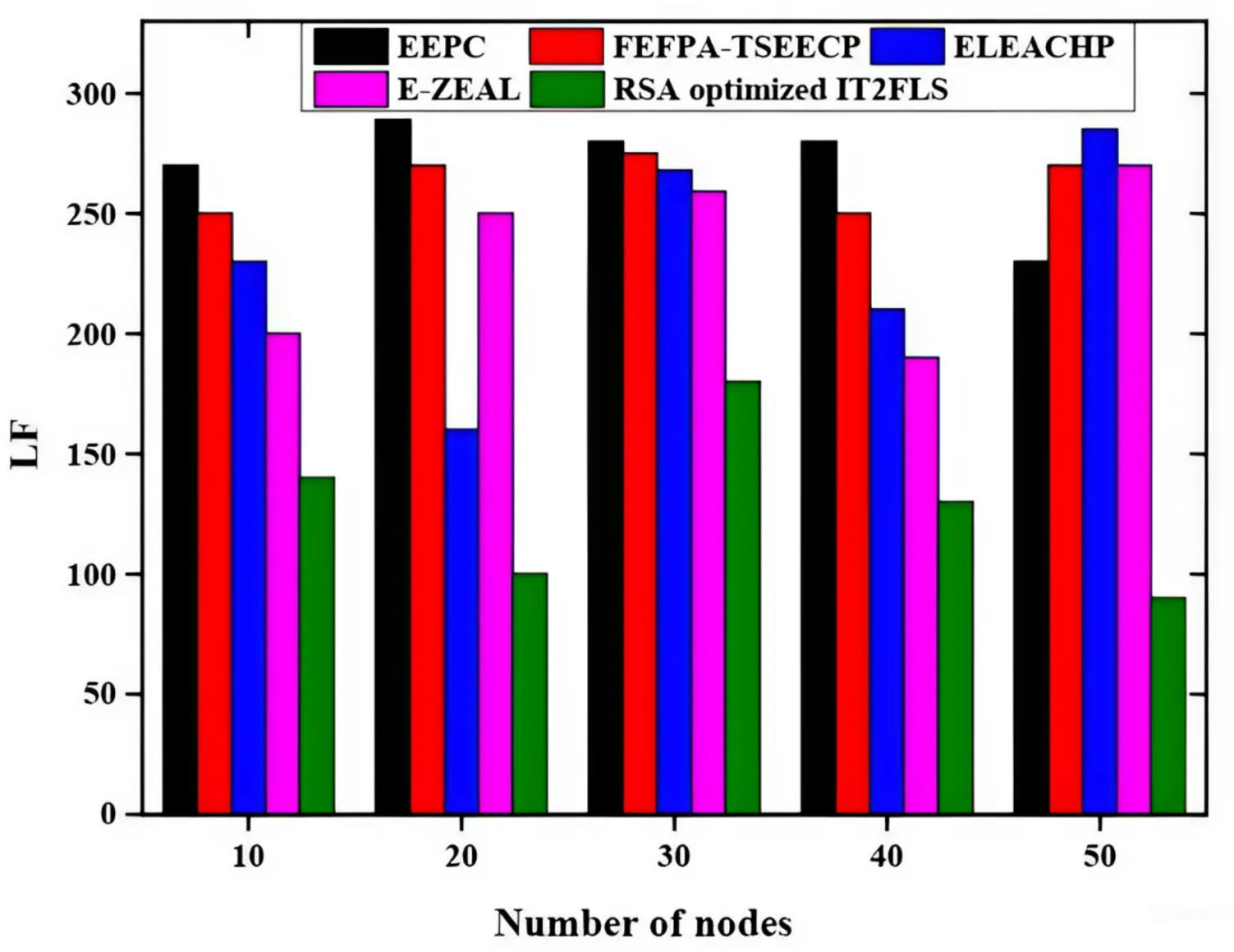
Comparative analysis of load factor.

Figure 5 describes the throughput of a number of nodes and the methods like EEPC, FEFPA-TSEECP, ELEACHP, E-ZEAL, and RSA optimized IT2FLS are used for obtaining the best throughput values. The proposed RSA optimized IT2FLS method gives high throughput values in packet transmission. The throughput value is rapidly increased with comparing the proposed RSA optimized IT2FLS method with other methods involves analysing the number of nodes used in the algorithms, RSA optimized IT2FLS method has a very high throughput value. Figure 6 shows the load factor (LF) using various methods like EEPC, FEFPA-TSEECP, ELEACHP, E-ZEAL^36^, and RSA optimized IT2FLS which is employed for giving excellent results in load factors. The RSA optimized IT2FLS method has very low load factors compared with other methods. In the network, the delay of the RSA-optimized IT2FLS method is reduced with respect to load factors.

**Fig. 7:**
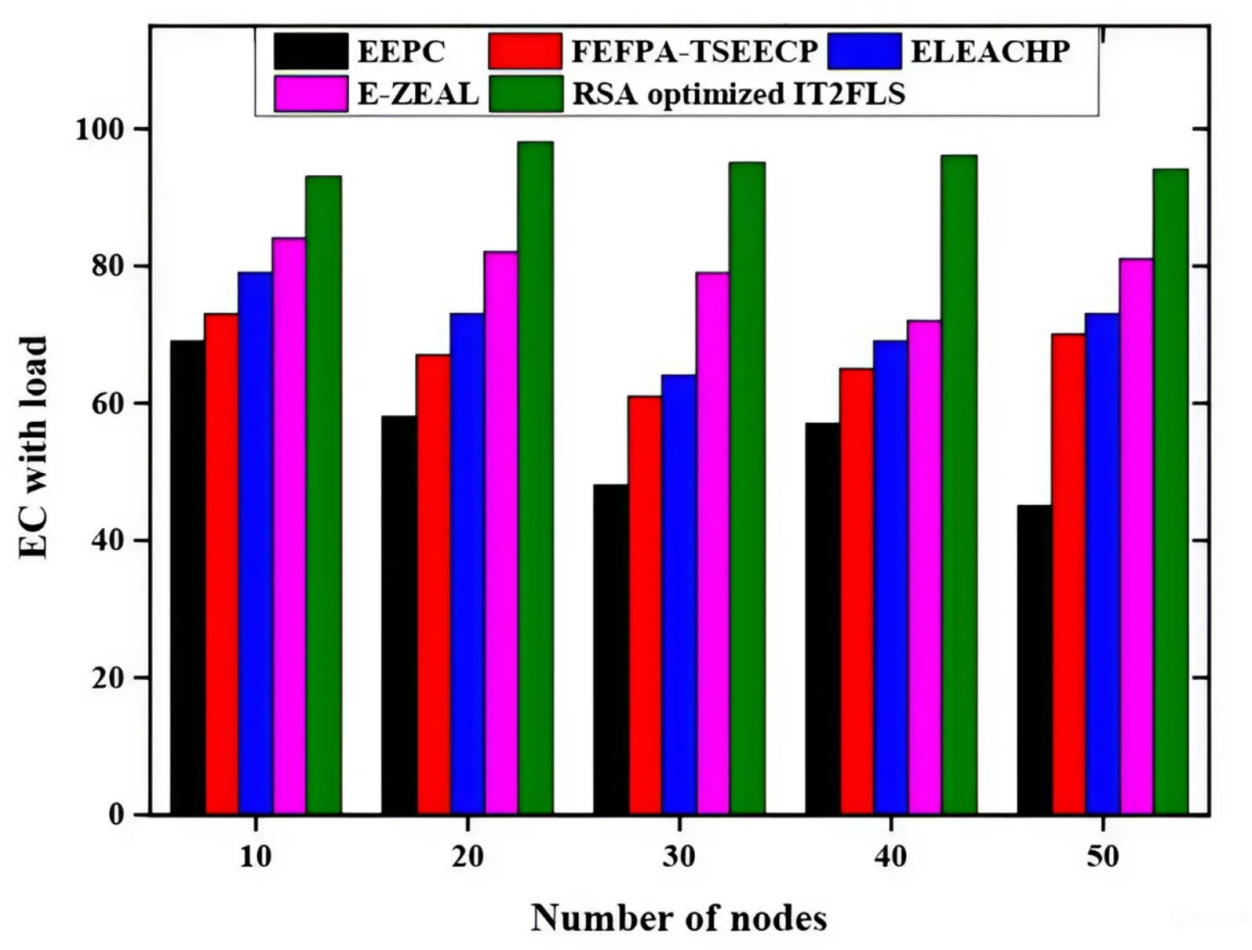
Comparative analysis of energy consumption with load.

**Fig. 8:**
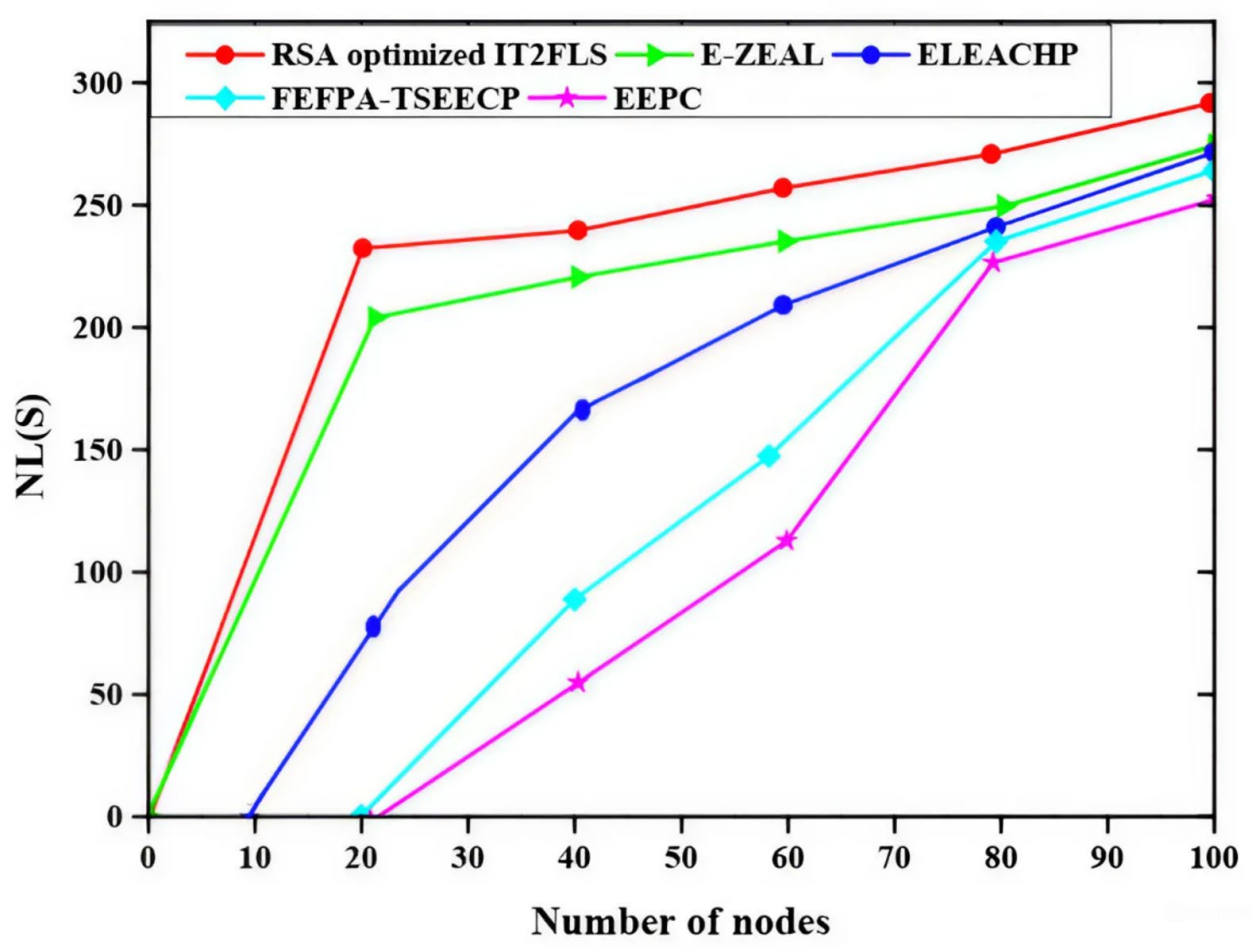
Comparative analysis of network lifetime.

Energy consumption (EC) with respect to load is represented in Fig. 7 and the different methods such as EEPC, FEFPA-TSEECP, ELEACHP, E-ZEAL, and RSA optimized IT2FLS are applied to achieve the best results. When the load factor is applied to the network, Energy consumption grows proportionally with the load factor. Compared to other methods, the energy usage relative to the RSA load optimized IT2FLS method has a very high value Fig. 8 represents network lifetime (NL) of various methods such as EEPC, FEFPA-TSEECP, ELEACHP, E-ZEAL, and RSA optimized IT2FLS are utilized for the best values. Increasing the number of nodes results in a longer network lifetime and energy consumption is decreased at the same time^37^. Three network lifetime of the RSA optimized IT2FLS method has a very high value compared with other methods.

**Fig. 9:**
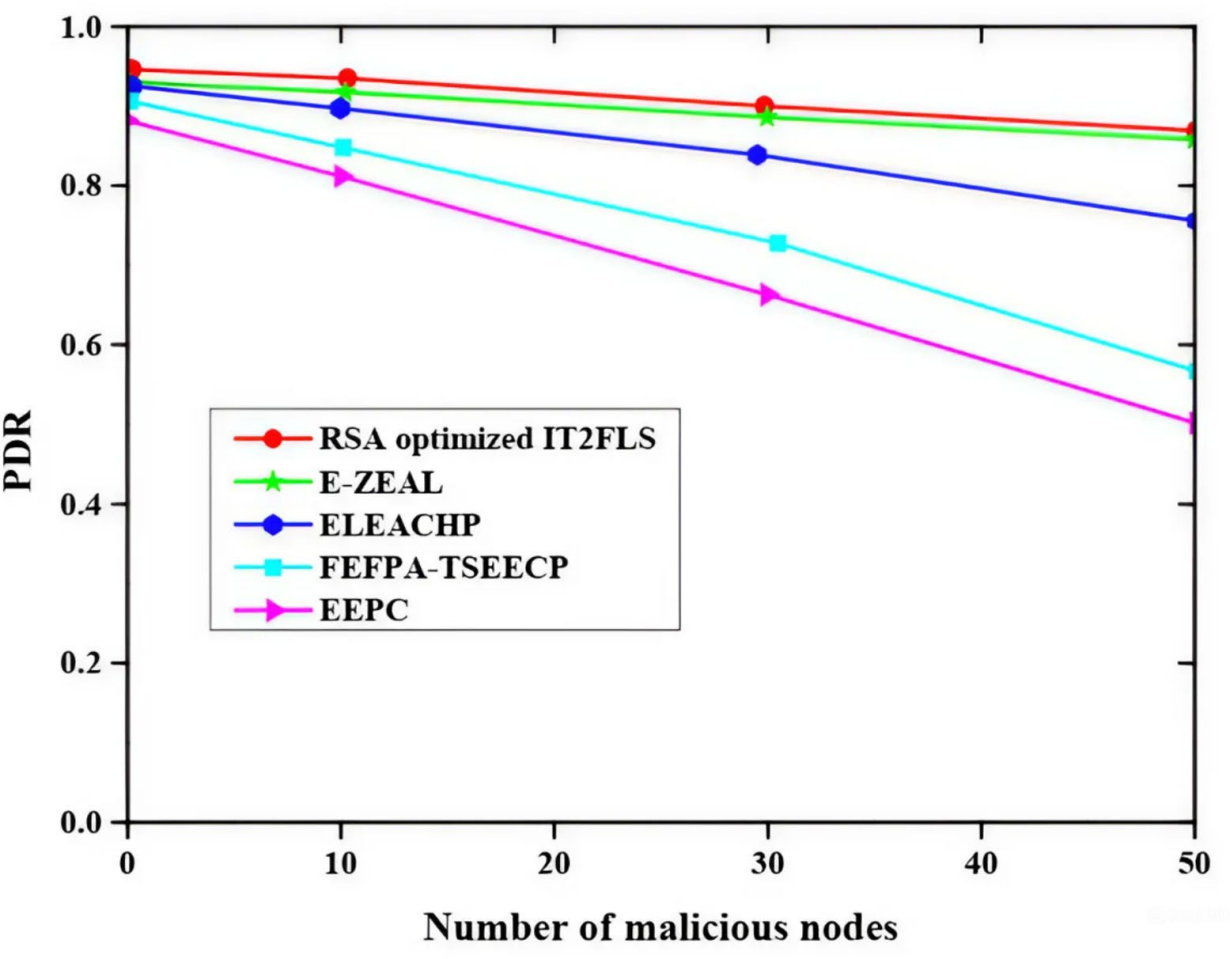
Effect of malicious nodes on packet delivery ratio.

Figure 9 shows the impact of different malicious nodes on packet delivery ratio (PDR) using different methods like EEPC, FEFPA-TSEECP, ELEACHP, E-ZEAL, and RSA optimized IT2FLS to analyze the effect. The rise in the quantity of malicious nodes leads in decrease in the packet delivery ratio. Among all those methods, the proposed RSA optimized IT2FLS method has a high packet delivery ratio.

**Fig. 10:**
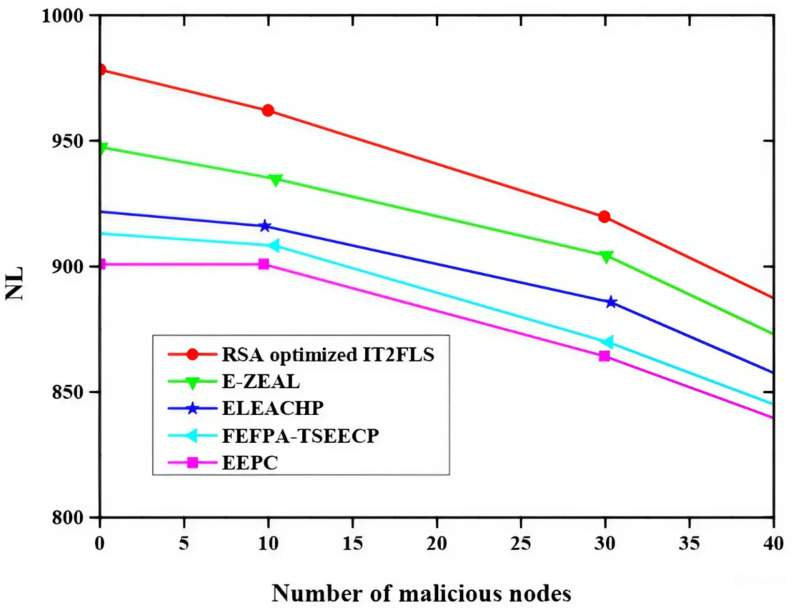
Effect of malicious nodes on network lifetime.

Malicious nodes effect on network lifetime (NL) with different methods such as EEPC, FEFPA-TSEECP, ELEACHP, E-ZEAL, and RSA optimized IT2FLS are described in Fig. 10. The proposed RSA optimized IT2FLS method has to select the best nodes for increasing the network lifetime. As the number of malicious nodes increases, the network lifetime automatically decreases^38^. The graphical representation shows that the RSA optimized IT2FLS method has a significantly longer network lifetime than other methods.

**Fig. 11:**
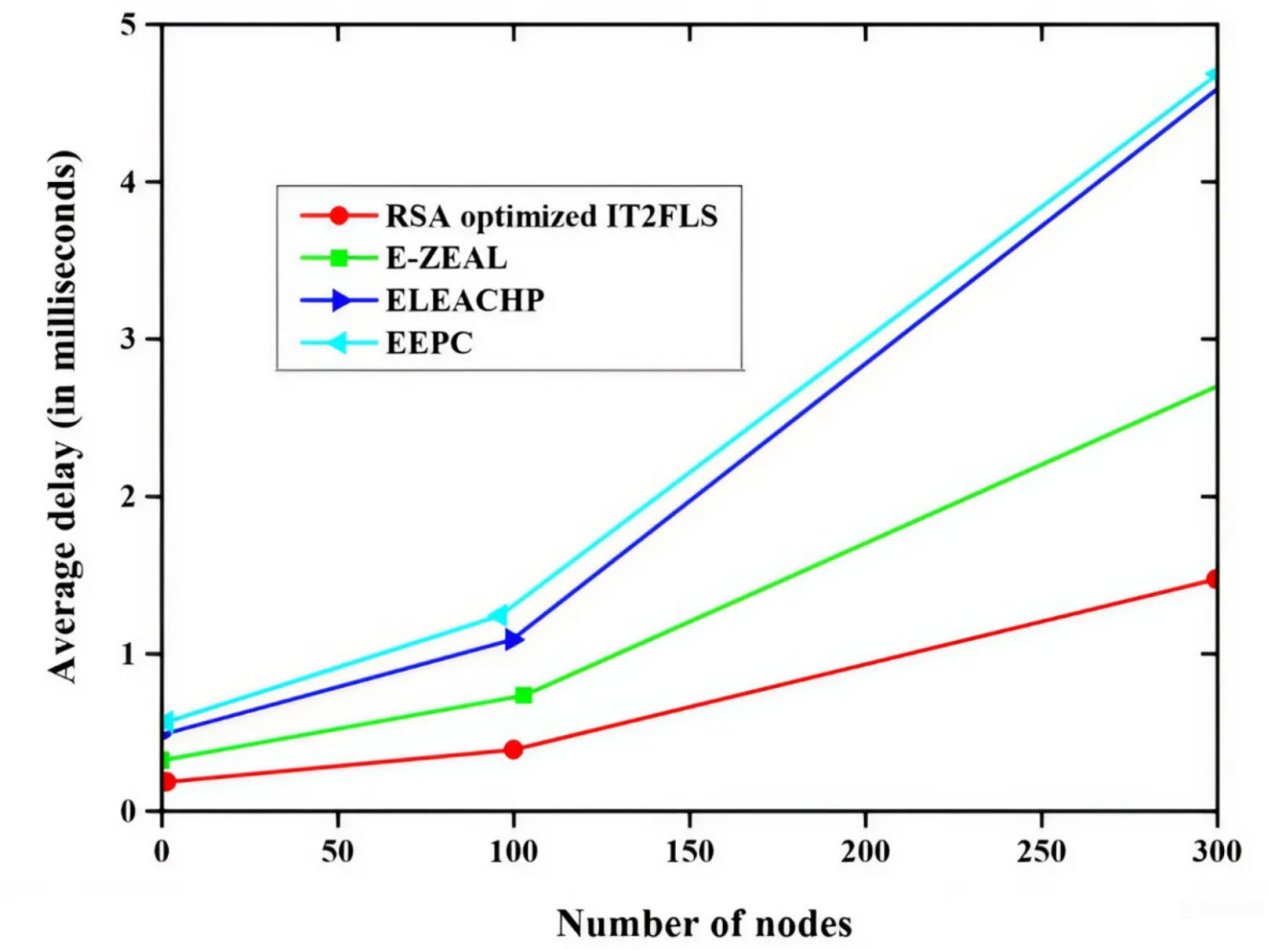
Node density effect on the delay value.

**Fig. 12:**
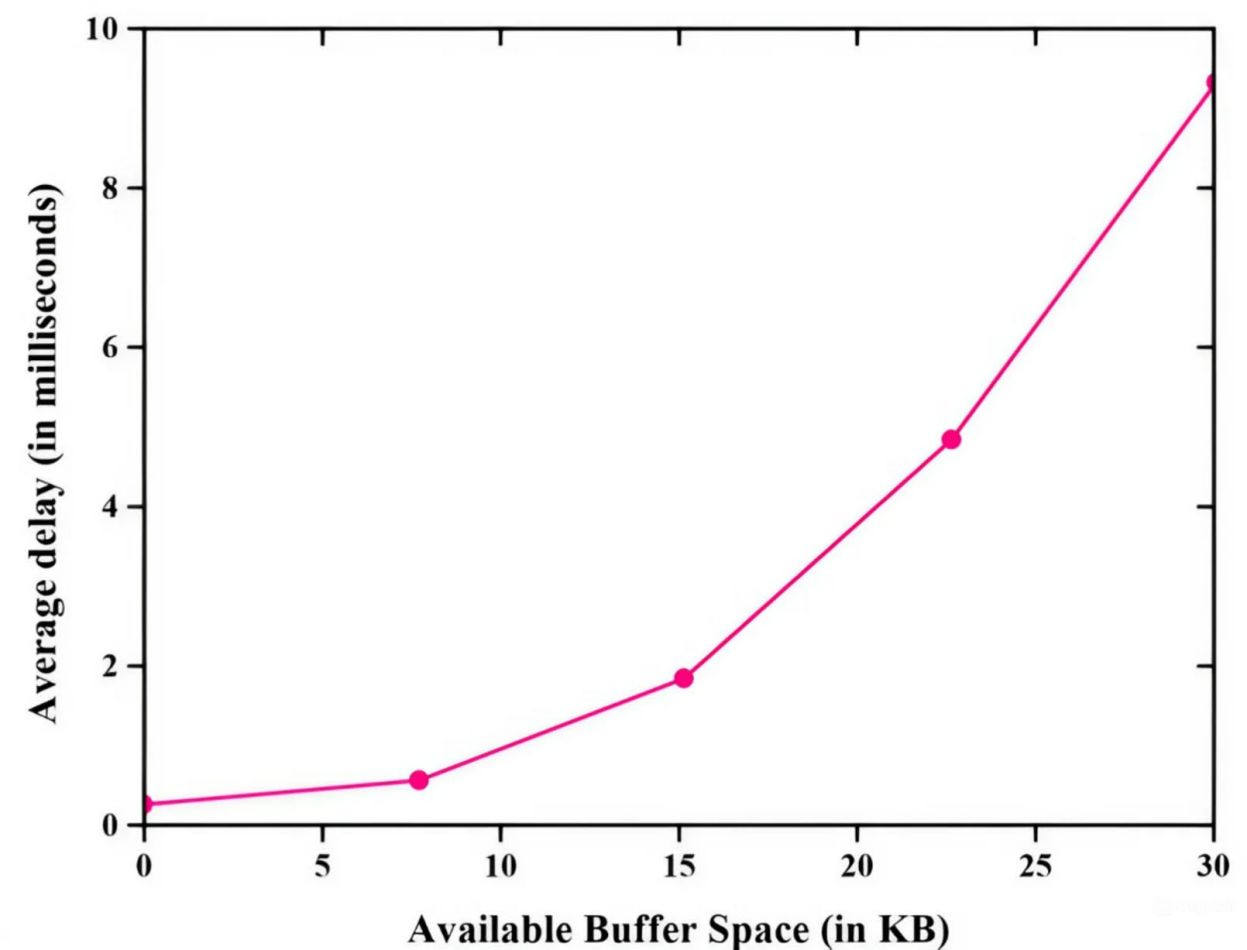
Availability of buffer space effect on the delay value.

The average delay performance of the method suggested can be seen in Fig. 11^39^. In the network, the delay increases along with the nodes. The delay in EENC is calculated using the distance between the communication nodes. For any two communication nodes, the delay value is constant. Figure 12 shows the buffer space availability that depends on queuing delay. This study demonstrates the potential of the Reptile Search Algorithm (RSA) optimized Type-2 Fuzzy Logic System (IT2FLS) in improving energy efficiency and QoS parameters in wireless sensor networks (WSNs). However, it currently lacks real-world implementation and validation, raising concerns about practical feasibility. Future research will prioritize field tests to assess the system’s adaptability and convergence behaviour in dynamic environments, thereby enhancing our understanding of its effectiveness across various operational contexts. The delay average is increased along with the availability of buffer space^40^. A node that is present in the packet can increase the average delay because the biggest buffer may enqueue more packets in it. The method suggested shows better performance compared to current methods. Because it avoids the neighbors which have a higher delay value and identifies the path with a smaller number of nodes. Interestingly, our proposed method has lower latency compared to EENC.

## Conclusions

This paper presents an Interval Type-2 Fuzzy Logic System (IT2FLS) model that significantly improves the quality of service (QoS) in secure routing for wireless sensor networks (WSNs), increasing the network lifetime. Achieving a dramatic 98% and 80% improvement in packaging delivery rates while significantly reducing energy consumption and end-to-end Using a hybrid clustering process that uses weighted ensembles and explicit ensemble selection for selecting the most appropriate cluster heads. The proposed model ensures efficient data collection and secure communication between sensor nodes. Performance evaluations based on established routing protocols such as EEPC, FEFPA-TSEECP, ELEACHP, E-ZEAL, and RSA-optimized IT2FLS demonstrate their superiority in increasing overall network performance. This study makes a significant contribution to the academic discourse on safe traffic in WSNs by providing a robust framework for the development of smart city infrastructure containing real-time safe and efficient information. This is essential for proper management of city resources. Additionally, future research can explore the integration of advanced machine learning techniques to improve the performance of routing protocols. and explore design changes that combine IT2FLS with other improvement techniques. to increase resilience to network complexity and security threats. Therefore, the demand will increase and meet the increasing demand for Reliable communication in complex technological environments.

## Data Availability

The labelled datasets used to support the findings of this study are available from the corresponding author upon request.
